# Galactose-modified small molecule modulator targets RORα to enhance circadian rhythm and alleviate periodontitis-associated alveolar bone loss

**DOI:** 10.1038/s41413-025-00445-w

**Published:** 2025-10-30

**Authors:** Guangxia Feng, Zhiwen Liao, Yifan Wang, Qingming Tang, Nayun Li, Cheng Li, Yuqing Liu, Renlong Liu, Mingjian Cui, Wenjie Fan, Ying Yin, Lingkui Meng, Jing Zeng, Zetao Chen, Guanzheng Luo, Peng Xiang, Qian Wan, Lili Chen

**Affiliations:** 1 Department of Stomatology, Union Hospital, Tongji Medical College, Huazhong University of Science and Technology, Wuhan, Hubei China; 2 School of Stomatology, Tongji Medical College, Huazhong University of Science and Technology, Wuhan, Hubei China; 3 Hubei Province Key Laboratory of Oral and Maxillofacial Development and Regeneration, Wuhan, Hubei China; 4 Hubei Key Laboratory of Natural Medicinal Chemistry and Resource Evaluation, School of Pharmacy, Huazhong University of Science and Technology, 13 Hangkong Road, Wuhan, Hubei China; 5 Hospital of Stomatology, Guanghua School of Stomatology, Sun Yat-sen University, Guangdong Research Center for Dental and Cranial Rehabilitation and Material Engineering, Guangzhou, China; 6 School of Life Sciences, Sun Yat-sen University, Guangzhou, China

**Keywords:** Bone, Dental diseases

## Abstract

Circadian rhythm disorders are associated with dysfunction in inflammatory diseases, and targeted regulation of the circadian rhythm could serve as an intervention strategy. RORα/γ, as core components of circadian clock genes, positively modulate the key circadian molecule BMAL1. In this study, Gala-SR, a potent small-molecule compound designed to effectively regulate circadian rhythms, was synthesized through a monosaccharide modification prodrug strategy via a hydrolysable conjugation of galactose onto SR1078, an unique synthetic agonist of RORα/γ. Compared with SR1078, Gala-SR exhibited significantly greater aqueous solubility, cytocompatibility, pharmacokinetic characteristics and efficacy in the targeted activation of RORα. Importantly, Gala-SR ameliorated rhythm disorders by enhancing amplitude of the circadian rhythm both in vitro and in vivo. In circadian rhythm disordered mice with periodontitis, Gala-SR restored local circadian rhythm and mitigated inflammation in periodontal tissue in a circadian clock-dependent manner, and alleviated alveolar bone loss. Our study demonstrates that Gala-SR exhibits great promise in restoration of circadian rhythm and could potentially serve as a targeted therapeutic intervention for treating inflammatory diseases arising from disruptions in circadian rhythm. This work provides a feasible paradigm for the development and translational application of small molecule modulators targeting circadian rhythms.

## Introduction

The circadian rhythm, which is ubiquitously observed in various biological behaviors and physiological activities,^1,2^ such as sleep-wake cycle, food intake, blood glucose regulation, cell differentiation and cell metabolism,^3^ is an evolutionary result progressively established by organisms to effectively acclimate to the changing environment.^4^ The generation, maintenance, and regulation of circadian rhythm fundamentally depend on an intrinsic circadian clock gene system, which consists of a core oscillator that precisely regulates its own transcriptional and translational activities via multiple feedback loops.^5^ The central circuit comprises both positive elements (transcriptional activators CLOCK/BMAL1 or NPAS2/BMAL1) and negative elements (PER1/2 and CRY1/2) that are responsible for generating molecular rhythms, whereas competing nuclear receptors REV-ERBs and RORs with opposite effects regulate BMAL1 expression to confer rhythm stability and robustness.^6^ Circadian rhythm disorders are implicated in metabolic syndrome, cardiovascular disease,^7^ cancer,^8^ immune system disorders,^9^ bone development retardation,^10^ and periodontitis.^11^ Accumulating evidence demonstrates that circadian rhythm disturbances significantly contribute to the pathogenesis and progression of periodontitis, the bidirectional relationship between circadian regulation and periodontal homeostasis has gained substantial attention, with emerging research revealing that circadian clock genes play pivotal roles in modulating inflammatory cascades and bone remodeling processes in periodontal disease.^12^ Recent studies have highlighted the therapeutic potential of small-molecule circadian rhythm modulators in ameliorating circadian-related disorders.^13^ Growing evidences show that small molecule modulators for circadian rhythms can be utilized to improve circadian disorders.^14^ One such modulator is melatonin, which is well-known for its ability to improve sleep and reverse jet lag.^15^ However, systemic application of melatonin has been reported to produce various adverse effects, including increased risk of stroke, endocrine disorders, and infertility.^16^ This is due to the multifaceted nature of melatonin as a hormone, as it exerts pleiotropic effects on various physiological systems.^17^ The small molecules that specifically target the component of the core oscillator are likely to exhibit less side effects other than its circadian regulatory functions.^16^ RORα, a member of the RORs family, acts as a pivotal positive regulator of BMAL1 transcription, thereby maintaining the balance of the RORα/REV-ERBα/Bmal1 core feedback loop.^18^ Empirical researches have corroborated that RORα contributes to the preservation of circadian rhythm, concurrently exerting anticancer,^19^ anti-inflammatory,^20^ and metabolic homeostasis maintenance for the sake of overall organismal health.^21^ Furthermore, RORα demonstrates high susceptibility to small-molecule modulators, rendering it a promising therapeutic target for circadian rhythms disorders and related diseases. Dozens of small molecule modulators of RORα, including natural ligands such as noruscogenin, nobiletin,^22^ cholesterol, and its derivatives have been discovered in the past two decades.^23^ Highly potent and selective synthetic ligands were further developed on the basis of analyzing the crystal structures of the complex between RORα and these natural ligands. By targeting RORα, these synthetic modulators have demonstrated efficacy in inhibiting autoimmunity,^24^ and reducing blood glucose in diabetic mice.^25^ However, the exact influence of these small-molecule modulators targeting RORα in ameliorating circadian rhythm-related inflammatory diseases remains to be elucidated.

**Fig. 1:**
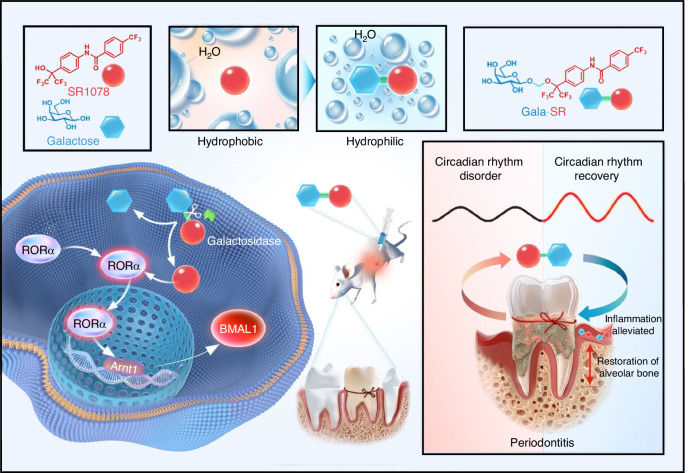
The designing strategy and working principle of the small molecular circadian rhythm modulator, Gala-SR, involve the conjugation of SR1078 with galactose via a self-immolative acetal linker. This modification endows Gala-SR with significantly improved aqueous solubility, cytocompatibility, and pharmacokinetic characteristics. Gala-SR is susceptible to cleavage by galactosidase, which liberates SR1078 to activate RORα, thereby enhancing the amplitude of circadian rhythm both in vitro and in vivo. The administration of Gala-SR effectively restores the local circadian rhythm of periodontal tissue and markedly reduces alveolar bone loss in circadian rhythm disordered mice with periodontitis

The limited aqueous solubility of these modulators, which is may inducing local cell apoptosis, poses a significant barrier to their clinical translation. Beyond solubility challenges, their functional efficacy and translational potential are further compromised by its suboptimal cell permeability, metabolic stability, and tissue compatibility. Water solubility stands as the primary issue, with other potential effects likely arising from this fundamental limitation. Prodrug design is a common and effective strategy in screening small molecule drugs.^26^ By covalently incorporating specific functional groups or carrier molecules at the proper site, it is feasible to optimize the physiochemical properties of small molecule compounds.^27^ The prodrug can release the active ingredient through enzyme or non-enzyme mediated activation based on known biological transformations to exert the pharmacodynamic efficacy.^26^ Saccharide conjugation, particularly the monosaccharide or disaccharide, as a convenient strategy in prodrug design, facilitates the enhancement of water solubility, biological activity, and in vivo stability while mitigating toxic side effects.^28^ By employing a self-immolative linker (SIL), the conjugated saccharide molecules can be cleaved through the enzymatic hydrolysis of glycosidases to liberate the active compound.^29^ Moreover, the incorporation of monosaccharides, such as glucose and galactose enable the exploitation of sodium glucose cotransporters for facilitated diffusion,^30^ efficiently promoting the absorption of drug molecules.^31^ For instance, glycosylated flavonoid compounds are recognized for their enhanced solubility and improved bioavailability compared with their non-glycosylated analogs.^32^ In light of this, we proposed to use SR1078,^33^ the only commercialized synthetic agonist of RORα identified thus far, as a representative example to systematically investigate the impact of saccharide modification on its aqueous solubility, pharmacokinetic profiles, in vitro and in vivo toxicity, as well as its efficacy in targeted modulation of RORα. After comprehensive comparison of three different saccharide modified compounds, the galactose modified SR1078, namely, Gala-SR, was chosen for further in-depth research exploring the precise impact on the cyclic expression of BMAL1 and the circadian rhythm in mice. Furthermore, given that circadian rhythm disorders were found to exacerbate the onset and progression of periodontitis,^34,35^ a precursory translational study was conducted to investigated the therapeutic efficacy of Gala-SR on alveolar bone resorption in periodontitis mice with circadian rhythm disorders (Fig. 1).

## Results

### The Galactose modification of SR1078 markedly improved the water solubility, cytocompatibility, pharmacokinetic characteristics and targeted activation efficacy toward RORα

**Fig. 2:**
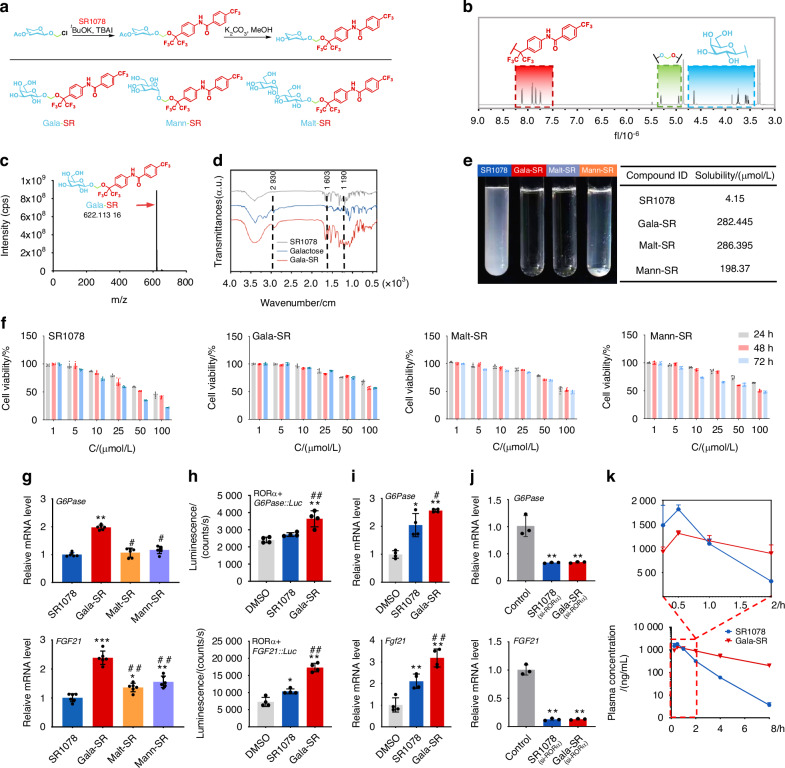
The fabrication, characterization, and functional evaluation of saccharides modified SR1078. **a** The principle of SR1078 modification; **b** ^1^H NMR; **c** MS; **d** FTIR spectra of Gala-SR; **e** Aqueous solubility characterization of SR1078, Gala-SR, Malt-SR, and Mann-SR. The left panel shows their aqueous solutions with a concentration of 10 mmol/L in DMSO, while the right panel is their kinetic solubility at 25 °C, respectively; **f** CCK-8 assay of human U2OS cells treated with SR1078, Gala-SR, Malt-SR, and Mann-SR at 1, 5, 10, 25, 50, and 100 μmol/L for 24, 48, and 72 h (*n* = 6); **g** HepG2 cells treated with SR1078, Gala-SR, Malt-SR, and Mann-SR at 5 μmol/L for 24 h, followed by PCR analysis to assess the mRNA expression levels of the downstream target genes *G6Pase* and *FGF21* by RORα/γ (*n* = 6, *represents the comparison with SR1078, and ^#^represents the comparison with Gala-SR, **P <0.01, ***P <0.001, ^#^*P* <0.05, ^##^*P* <0.01); **h** The luminescence of HEK293T cells cotransfected with RORα and a reporter consisting of the *G6Pase* or *FGF21* promoters upstream of a luciferase reporter gene upon addition of 5 μmol/L SR1078 and Gala-SR (*n* = 4); **i** The relative mRNA expression of *G6Pase* and *Fgf21* in liver tissue collected 24 h after intraperitoneal injection of DMSO, SR1078, and Gala-SR at a dose of 10 mg/kg in mice (*n* = 4); **j** The relative mRNA expression of *G6Pase* and *FGF21* in HEK293T cells treated by of SR1078 and Gala-SR at 5 μmol/L for 24 h after knocking down RORα with the siRNA plasmid targeting RORα (*n* = 3), (* represents the comparison with DMSO and # represents the comparison with SR1078, *P < 0.05, **P < 0.01, ^#^*P* < 0.05, ^##^*P* < 0.01). **k** The plasma levels of SR1078 and Gala-SR following intraperitoneal injection of the compounds at a dose of 10 mg/kg in mice

SR1078 is currently the unique commercially available synthetic agonist of RORα/γ, and it exhibits excellent selectivity and activation capabilities.^33^ However, the aqueous solubility of SR1078 is much limited, severely constraining its potential applications and utilization methods. Insufficient solubility may lead to inadequate absorption, limited bioavailability, reduced systemic circulation concentration, diminished blood exposure, and compromised therapeutic efficacy.^34^ Monosaccharide or disaccharide conjugation is an effective prodrug strategy to improve the water solubility, cytotoxicity, and pharmacokinetic characteristics of compounds.^11,26^ Herein, three modified agonists of RORα/γ, namely, Gala-SR, Malt-SR, and Mann-SR, were synthesized through the introduction of galactose, maltose, and mannose moieties via acetal linker formation on the hydroxyl group of SR1078 (Fig. 2a). Nuclear magnetic resonance spectra of hydrogen, carbon, and fluorine (^1^H NMR, ^13^C NMR, and ^19^F NMR) (Fig. 2b, Fig. S1–24), as well as mass spectra (MS) analysis (Fig. 2c) demonstrated successful synthesis. Fourier transform infrared (FTIR) spectra further confirmed the fabrication of Gala-SR, as the results showed that the peaks corresponding to benzen ring skeleton vibration at approximately 1 603 cm^−1^ and CF_3_ stretching vibration at about 1 190 cm^−1^, which belonged to SR1078, and the stretching vibration peak of alkyl groups on saccharide ring at 2 930 cm^−1^ that came from galactose, were all presented in Gala-SR (Fig. 2d). After modification, the kinetic water solubility markedly increased from 4.15 μmol/L for SR1078 to 282.445 μmol/L for Gala-SR, 198.37 μmol/L for Mann-SR, and 286.395 μmol/L for Malt-SR, showing a 50–70-fold improvement (Fig. 2e). This result indicated that monosaccharide and disaccharide conjugation are indeed effective ways to improve the water solubility of the agonist. However, given that galactose, mannose, and maltose have water solubilities of 680 g/L, 2 480 g/L, and 64 g/L at room temperature, respectively, the water solubility of saccharide molecules may not directly determine the water solubility of the modified agonist molecules.

Through the live-dead staining and the CCK8 assay, our investigation revealed that all three modified small molecules exhibited reduced cytotoxicity relative to SR1078, that being particularly pronounced at concentrations exceeding 10 μmol/L (Fig. 2f and Fig. S25). Notably, the cell compatibility of Gala-SR was identified as superior among all the evaluated groups. The body weight changes in mice intraperitoneal injected with the compounds further confirmed a reduced systematic toxicity of Gala-SR compared to SR1078, particularly at a higher dose of 50 mg/kg (Fig. S26). As a commercialized small molecule agonist, SR1078 has been reported to have a safe dose of 5 μmol/L for in vitro experiments and 10 mg/kg for animal studies in mice.^33^ As evident from the aforementioned results, the galactose modification significantly broadened the safety dosage range of SR1078. In order to explore the influence of different saccharides moiety modifications on the activation of RORα/γ by SR1078, we conducted a transcriptional analysis of two direct target genes of RORα/γ, namely, glucose-6-phosphatase (*G6Pase*) and fibroblast growth factor-21 (*FGF21*).^33^ The result showed that Gala-SR induced a significant upregulation of both *G6pase* and *FGF21* expression in HepG2 cells compared to SR1078, with an effect that surpassed those observed with Malt-SR and Mann-SR (Fig. 2g). The above findings indicate that among three glycosylated molecules, galactose demonstrated superior performance in enhancing the water solubility, cellular compatibility, and RORα/γ activation efficacy of SR1078. Therefore, Gala-SR was selected for the following research.

A luciferase reporter gene assay was further conducted in HEK293T cells co-transfected with a full-length RORα gene and a luciferase reporter gene driven by *G6Pase* promoter and *FGF21* promoter, respectively. Following co-transfection, treatment with 5 μmol/L of both SR1078 and Gala-SR manifested a marked augmentation in the luminescence of both the *G6pase* and *FGF21* groups. More importantly, Gala-SR elicited a substantially elevated expression of the *G6pase* and *FGF21* luciferase reporter genes compared to SR1078 (Fig. 2h). Given that SR1078 also activates RORγ, we further co-transfected RORγ gene with *G6pase* or *FGF21* luciferase reporter gene in HEK293T cells. Results demonstrated that both Gala-SR and SR1078 significantly enhanced the luminescence intensity of *G6pase* and *FGF21* reporter genes compared to DMSO, while no significant difference was observed between them. The findings presented herein suggest that the galactose modification of SR1078 significantly enhances its targeted activation efficacy towards RORα selectively, particularly at a concentration of 5 μmol/L (Fig. S27a). Interestingly, such significant enhancement of SR1078 over Gala-SR in promoting RORα activation was attenuated at higher concentrations (Fig. S27b). The superiority of Gala-SR in activating RORα was further confirmed in vivo, wherein Gala-SR significantly upregulated *G6Pase* and *FGF21* expression in mice liver compared to SR1078 (Fig. 2i). Notably, the knockdown of RORα markedly hampered the activation of both Gala-SR and SR1078 in the transcription of *G6Pase* and *FGF21* genes (Fig. 2j and Fig. S27c).

### The Galactose modification of SR1078 markedly improved pharmacokinetic characteristics

We subsequently examined the pharmacokinetic properties of Gala-SR and SR1078 in mice through intraperitoneal (IP) administration. We noted a typical in vivo burst release within 0.5 h after a single injection of 10 mg/kg in both groups. Nevertheless, Gala-SR exhibited significantly prolonged in vivo retention compared to that of SR1078. Eight hours after injection, the mean plasma concentration of SR1078 sharply decreased to 2.96 ng/mL, whereas Gala-SR maintained a mean plasma concentration of 202 ng/mL 8 h after injection (Fig. 2k). The mean retention times (MRTs) of SR1078 and Gala-SR were calculated to be 1.19 h and 4.50 h, respectively (Fig. S28, Table S1). To evaluate the bioavailability, we also conducted the intravenous (IV) administration. The area under the curve (AUC) (h*ng/mL) of Gala-SR-IV, Gala-SR-IP, SR1078-IV, and SR1078-IP was 8 176.18, 6 077.36, 2 542.41, and 1 909.62, respectively (Table S1). The bioavailability of Gala-SR and SR1078 can be calculated as approximately 74.3% and 75.1%, respectively, showing no significant difference. Nevertheless, this approach still demonstrated a substantial enhancement in therapeutic efficacy and optimization of pharmacokinetic characteristics.

### The hydrolysable galactose modification determines the efficacy of Gala-SR targeted activation on RORα

**Fig. 3:**
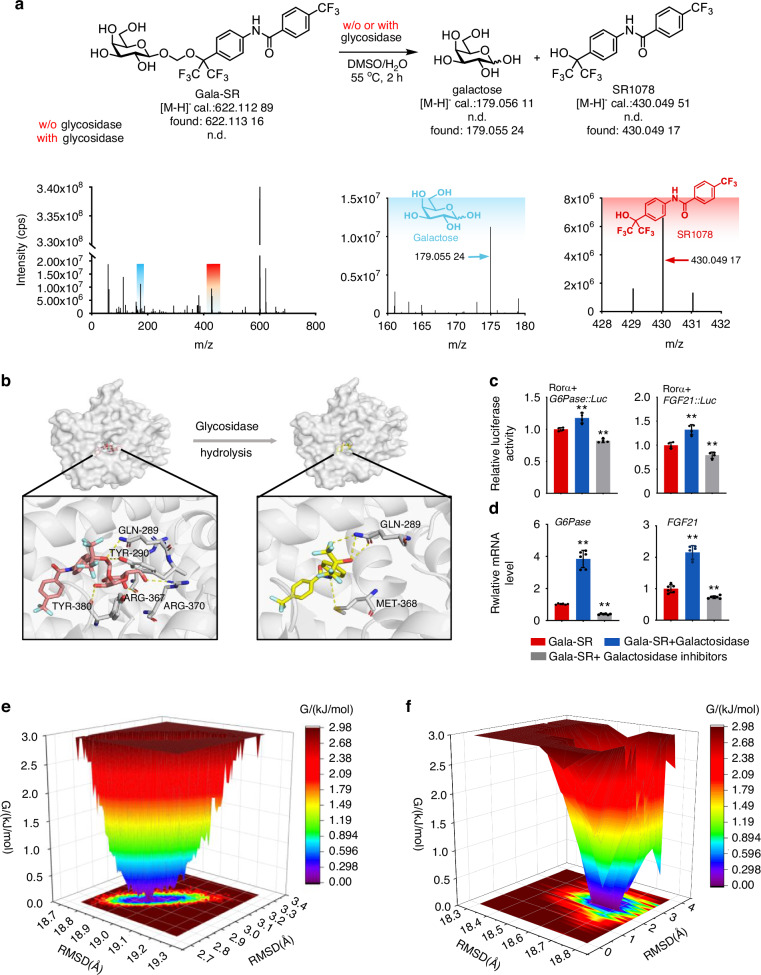
Exploring the impact of acetal linker design on the targeted regulatory function of RORα. **a** The small molecules galactose and SR1078 are generated upon the cleavage of Gala-SR by β-galactosidase; The upper part: a schematic diagram illustrates the process of enzymatic hydrolysis. Lower part: MS spectra of Gala-SR after hydrolysis by β-galactosidase; **b** Binding pose of Gala-SR and SR1078 on the simulated RORα structure predicted by AutodockVina; **c** The luminescence of HEK293T cells cotransfected with RORα and a reporter containing the G6Pase or FGF21 promoters upstream of a luciferase reporter gene was measured following the addition of 5 μmol/L Gala-SR and combined with β-galactosidase or β-galactosidase inhibitor (*n* = 4); **d** The mRNA expression of *G6Pase* and *FGF21* in HepG2 cells treated with 5 μmol/L of Gala-SR and combined with β-galactosidase or β-galactosidase inhibitor. (*n* = 6). (* represents the comparison with Gala-SR, **P < 0.01); **e**, **f** Molecular dynamics simulation of the interaction between Gala-SR, SR1078 and RORα

The acetal utilized in our study, serving as a SIL to conjugate galactose onto SR1078, has been previously documented to exhibit susceptibility towards enzymatic hydrolysis by endogenous β-glycosidases.^27^ The hydrolysis leads to the release of galactose, SR1078, and formaldehyde, an endogenous metabolite that undergoes conversion into formate to facilitate one carbon metabolism.^36^ The consistent results were found in our study as the mass spectrometry confirmed that the hydrolysis of galactose on, Gala-SR occurred upon the addition of galactosidase (Fig. 3a). The peak at 430.049 17  (m/z) also demonstrated the release of intact SR1078. To validate the impact of such hydrolysable prodrug design on the activation efficacy of Gala-SR toward RORα, we simulated the structural interactions between Gala-SR and RORα before and after the hydrolysis. Gala-SR interacts with RORα mainly through the formation of hydrogen bonds between the TYR-380, ARG-367, and ARG-370 of RORα and the hydroxyl groups on the galactose moiety (Fig. 3b). However, the SR1078 released after hydrolysis of Gala-SR forms hydrogen bonds with GLN-289 via fluoromethyl groups and with MET-368 via amino groups. These disparities lead to discernible structural variances in RORα, which potentially exert a significant influence on the transcriptional activation of downstream target genes by RORα. This assumption was evidenced by our finding, as a significantly enhanced expression of both G6Pase and FGF21 luciferase reporter genes was detected in HEK293T cells co-transfected with RORα (Fig. 3c, Fig. S29) when Gala-SR was combined with galactosidase. Conversely, when cells were treated with a galactosidase inhibitor, the effect of Gala-SR on the activation of each luciferase reporter was significantly decreased compared to the original level (Fig. 3c). Consistent results at the transcriptional level were verified in HepG2 cells, where the addition of galactosidase and galactosidase inhibitors significantly upregulated and downregulated the expression of *G6Pase* and *FGF21*, respectively, compared to those treated with pure Gala-SR (Fig. 3d).

Moreover, molecular dynamics simulation of Gala-SR with RORα revealed a predominant conformational cluster with RMSD values ranging from 2.8 to 3.0 Å and Rg values between 18.8 and 19 Å (Fig. 3e), whereas SR1078 with RORα showed RMSD values of 1.4−3.2Å and a smaller Rg values of 18.5−18.8 Å (Fig. 3f), this further demonstrating that the hydrolysable acetal linker designed herein is the key factor determining the efficacy of Gala-SR-mediated targeted activation of RORα.

### Gala-SR enhances circadian rhythm in vitro and in vivo

**Fig. 4:**
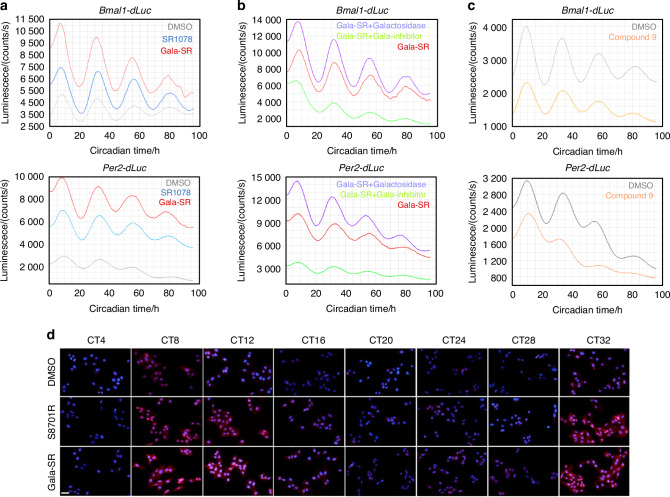
Gala-SR enhances circadian rhythm amplitude in vitro. **a** Bioluminescent recordings of *Per2-dLuc* and *Bmal1-dLuc* U2OS cells upon treatment of 2 μmol/L of SR1078 and Gala-SR while DMSO was set as control (*n* = 3); **b** Gala-SR, and Gala-SR combined with β-galactosidase (205 U/mg) or with β-galactosidase inhibitor (26 mmol/L) treatment (*n* = 3); **c** RORα-targeting inhibitor Compound 9 (5 μmol/L) with a control of DMSO (*n* = 3); **d** The immunofluorescence of BMAL1 at different time points in synchronized U2OS cells that were treated with 5 µmol/L of SR1078 and Gala-SR, and immunofluorescence was used to detect the expression of BMAL1 at different time points, the scale bar is 50 µm

The circadian rhythm regulated by the core clock gene *Bmal1* consists of three key parameters, period, phase, and amplitude, among which amplitude serves as an evaluative parameter for circadian rhythm robustness^36^. RORα is effectively activate the expression of BMAL1. However, the precise impact of its synthesized agonist SR1078 and saccharide-modified derivative Gala-SR on the circadian rhythm remains elusive. To this end, we detected the rhythmic expression of core circadian rhythm genes *Bmal1* and *Per2* after administering Gala-SR and SR1078 in U2OS cells that harbor a luciferase gene controlled by either the *Per2* or *Bmal1* promoter (*Per2-dLuc* or *Bmal1-dLuc* cells).^37^ The overall bioluminescence levels in both *Bmal1-dLuc* cells and *Per2-dLuc* cells were significantly elevated following treatment with SR1078 compared to the DMSO, and Gala-SR exhibited a superior efficacy than SR1078 (Fig. 4a). Importantly, SR1078 administration resulted in a significant augmentation of the fluctuation degree of bioluminescence intensity, which was further enhanced by Gala-SR. Meanwhile, neither SR1078 nor Gala-SR treatment induced any obvious alterations in the period or phase of the bioluminescence (Fig. 4a). These findings suggest that the modulation of circadian rhythms by SR1078 and Gala-SR is mediated through an enhancement in the expression level and amplitude of core circadian genes, rather than alterations in period and phase. Notably, the enhancing effect of Gala-SR on the amplitude of BMAL1 increases with the dose, whereas higher doses of SR1078 are difficult to further promote its efficacy (Fig. S30).

We noticed that the treatment of Gala-SR with β-glycosidase and β-glycosidase inhibitor resulted in respective augmentation and suppression in circadian rhythm amplitude (Fig. 4b). These findings align strongly with our previous efficacy assessment of modulators targeting RORα activation. Concurrently, we synthesized a RORα-targeting inhibitor with a chemical structure resembling SR1078 namely compound 9 (Fig. S31–33), based on the details provided in a published patent,^38^ as a control to confirm the potential role of RORα activation by SR1078 and Gala-SR in enhancing rhythm. The successful inhibition of RORα transcriptional activity by compound 9 (Fig. S34), and the subsequent downregulate the amplitudes, without disrupting the fundamental period or phase of the circadian rhythm (Fig. 4c), provides strong evidence to support our previous findings. Furthermore, the corroboration at the protein level through immunofluorescence detection of BMAL1 reinforced our conclusion, that Gala-SR and SR1078 enhance the expression of BMAL1 by targeted activation of RORα (Fig. 4d).

In addition to the U2OS model cells, we also treated synchronized human periodontal ligament stem cells with small molecules and examined key circadian clock genes including *BMAL1, PER1, PER2, CRY1, RORα,* and *NR1D1*. The results demonstrated that compared to DMSO and SR078, Gala-SR more effectively enhanced the rhythmic expression amplitude of *BMAL1, PER1,* and *PER2* (Fig. S35a), this finding consistent with the observations in the U2OS model cells. Furthermore, detection of RORα downstream target genes *FGF21* and *G6Pase* also confirmed that Gala-SR is able to promote circadian gene expression amplitude in periodontal ligament cells (Fig. S35b). These findings provide compelling evidence supporting the potential therapeutic application of Gala-SR in periodontal tissues.

**Fig. 5:**
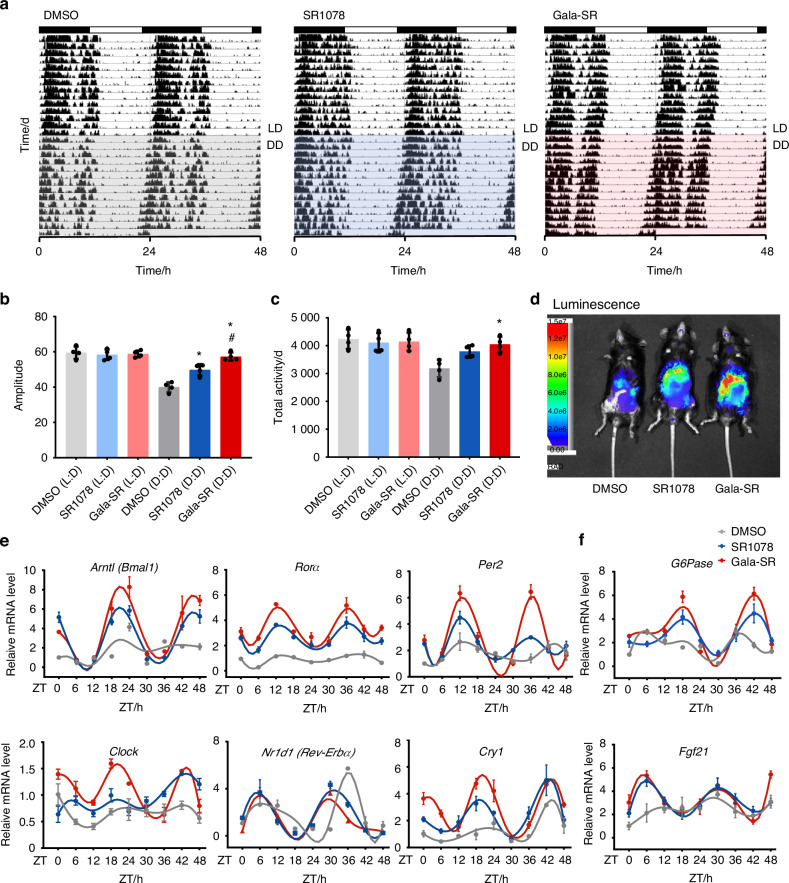
Gala-SR enhances the rhythm amplitude to improve circadian rhythms in vivo. **a** Actograms illustrating the effect of DMSO, SR1078 and Gala-SR on circadian behavior in C57 mice, SR1078 and Gala-SR (10 mg/kg) were intraperitoneally injected at ZT11 (*n* = 4); **b** Amplitude [*represents the comparison with DMSO (DD) and ^#^ represents the comparison with SR1078(DD), *P < 0.05, ^#^*P* < 0.05]; **c** Daily total activity of mice; **d** Live images of PER2::LUC mice that were intraperitoneally injected with SR1078, Gala-SR or DMSO at ZT0; **e** The mRNA expression of clock genes in liver tissues collected from jet lag mice treated with Gala-SR, SR1078, and DMSO at different time points (*n* = 4); **f** The mRNA expression of RORα downstream target gene in mice liver tissues at different time points (*n* = 4). (* represents the comparison with DMSO and ^#^represents the comparison with SR1078, *P < 0.05, ^#^*P* < 0.05, *n* = 4)

To investigate the potential impact of Gala-SR on the circadian rhythm in vivo, we monitored the circadian locomotor activity of mice under constant dark (D:D) conditions after 2 week of acclimation in a standard light:dark (L:D) setting by using a state-of-the-art small animal rhythm activity video monitoring system namely “Big Brother”.^36^ Under the L:D condition, all three groups of mice exhibited typical circadian rhythm characteristics, manifesting a complete activity-rest period lasting approximately 24 h. The rhythm patterns in mice demonstrated remarkable similarity across the groups (Fig. 5a). After exposure to the DD condition, no notable alterations were observed in the activity-rest circadian rhythm period in any of the mice. However, a slight forward shift in the activity phase was detected, without any significant difference among the three groups (Fig. 5b). Compared to DMSO, both SR1078 and Gala-SR significantly increased the activity rhythm (Fig. 5b) as well as total activity (Fig. 5c), with Gala-SR demonstrating a more pronounced efficacy.

To experimentally verify the effect of Gala-SR and SR1078 on circadian rhythm in mice, we imaged luciferase activity in PER2::LUC knock-in mice. We observed that liver luciferase signal was induced within 0.5 h of treatment with Gala-SR and SR1078, providing support for the conclusion that Gala-SR and SR1078 treatments could enhance circadian rhythm. Notably, Gala-SR administration resulted in more stronger luminescence signal (Fig. 5d). Thus, we further extracted the liver tissue of mice with disordered circadian rhythms and detected temporal changes in the expression of key circadian genes and the downstream target genes of RORα, including *Bmal1*, *Per2*, *Clock*, *Cry1*, *Rorα*, *Rev-Erbα*, *Fgf21* and *G6Pase*. The results demonstrated that Gala-SR effectively restored the circadian rhythmic in liver tissue, exhibiting the most profound increase BMAL1 (Fig. 5e), by targeted activation of RORα (Fig. 5f). Therefore, both the macroscopic observations of mouse circadian activity and microscopic alterations in the molecular circadian rhythm system within liver tissue collectively suggest that Gala-SR effectively enhances the amplitude of the animal circadian rhythm. Further assessment of blood biochemical indicators, including alanine aminotransferase (ALT), aspartate transaminase (AST), Creatinine (CREA), and blood urea nitrogen (BUN) did not reveal any significant differences among the three groups (Fig. S36a). Histopathological examination of liver, kidney, and heart, tissue sections following treatment with 10 mg/Kg Gala-SR revealed no discernible changes (Fig. S36b). Consistent with the previous finding in systemic toxicity evaluation by monitoring the mice body weight (Fig. S26), these results suggest that Gala-SR is of favorable biocompatibilities, effectively enhancing circadian rhythm amplitude in a safe manner in vivo.

### Gala-SR alleviates alveolar bone loss in circadian rhythm disordered mice with periodontitis

**Fig. 6:**
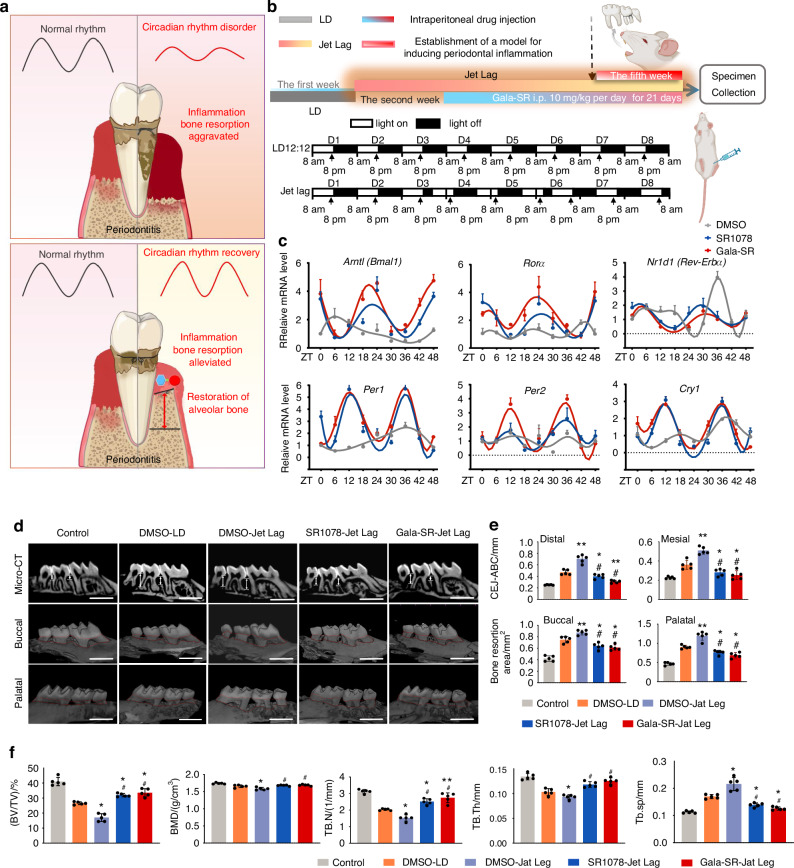
SR1078 and Gala-SR can improve alveolar bone resorption in mice with aggravated periodontitis due to circadian rhythm disruption. **a** Schematic diagram of the modeling process; **b** Schematic diagram of Gala-SR improving alveolar bone resorption in mice with periodontitis due to circadian rhythm disruption; **c** Gingival tissue was collected from mice treated with Gala-SR, SR1078, and DMSO in Jet lag. Real-time qPCR analyses were performed to determine mRNA expression of clock genes in mice periodontal tissues at different time points (*n* = 4); **d** Micro-CT(micro-computed tomography) and 3D reconstruction of the maxilla from mice treated with Gala-SR, SR1078, and DMSO, scar bars are 1 mm; **e** Comparison of bone resorption heights in the distal, mesial, buccal and palatal of the alveolar bone; **f** Comparison of the related bone histomorphometric parameters of the alveolar bone. (* represents the comparison with DMSO-LD and ^#^ represents the comparison with DMSO-Jet lag, *P < 0.05, **P < 0.01, ^#^*P* < 0.05, ^##^*P* < 0.01, *n* = 5)

Periodontitis has the highest incidence rate among all oral diseases and is a major cause of adult tooth loss. Previous epidemiological investigations have shown that individuals working night shifts have significantly exacerbated levels of periodontal tissue inflammation and alveolar bone resorption compared to those with normal work schedules.^39^ It is also reported that the inflammatory signaling pathways in periodontal tissue is over activated in periodontitis mice with circadian rhythm disorder.^40^ Given this, we have substantial grounds to hypothesize that the restoration of circadian rhythms could potentially ameliorate periodontitis. According to our assumption, periodontal inflammation in circadian rhythm-disordered mice is more critical than in control mice under L:D conditions, resulting in a more severe alveolar bone resorption. Administration of Gala-SR and SR1078 is expected to restore the circadian rhythm, thereby alleviating the inflammation and reducing the alveolar bone absorption (Fig. 6a). Hence, we established an animal model to evaluate the therapeutic efficacy of Gala-SR on alveolar bone resorption in periodontitis mice with circadian rhythm disorders.^41^ The mice were initially subjected to a one-week L:D feeding regimen, followed by exposure to disordered conditions for four weeks. At the beginning of the third week, the mice received intraperitoneal injections of Gala-SR or SR1078 (10 mg/kg) or the equivalent amount of DMSO utilized to dissolve these modulators every day for three consecutive weeks. Mice without any treatment and mice treated with DMSO but kept in L:D condition were also set for controls. By the end of the fourth week, a ligature-induced periodontitis model was established as previously described,^39^ and samples at different time points were collected one week later for subsequent tests (Fig. 6b).

The transcription levels of circadian genes including *Bmal1*, *Rorα*, *Rev-Erbα*, *Per1/2*, and *Cry1* in the periodontal tissue were unnaturally low and lost the circadian rhythmic features in jet lag mice treated with DMSO (Fig. 6c). Administration of SR1078 and Gala-SR restored their rhythmic expression and increased their overall expression level. Gala-SR further enhanced the circadian rhythm amplitude of *Bmal1*, *Per2*, and *Rorα*, compared with SR1078 (Fig. 6c). This result aligns with our previous findings in the mice liver, indicating a robust and consistent effect of Gala-SR in restoring the circadian rhythm and enhancing rhythm amplitude systemically throughout the body. Micro-CT analysis (Fig. 6d–f) revealed that the degree of alveolar bone resorption in mice with periodontitis was much worse in the DMSO-Jet Lag group than that in DMSO-LD group. These findings corroborate with the previous epidemiological reports, that circadian rhythm disorder aggravates periodontal inflammation and alveolar bone resorption.^40^ Compared to those treated with DMSO, circadian rhythm disordered mice injected with SR1078 and Gala-SR exhibited significantly reduced alveolar bone resorption, with Gala-SR showing a more prominent efficacy than SR1078 (Fig. 6d–f).

**Fig. 7:**
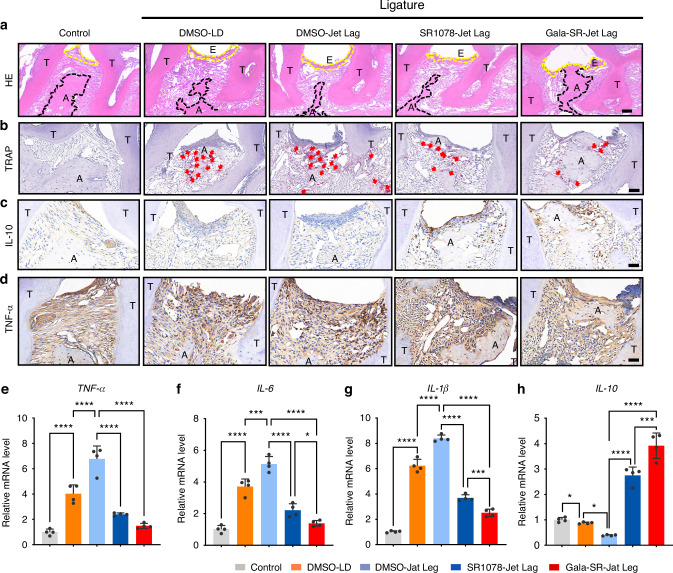
SR1078 and Gala-SR can improve inflammation in mice with aggravated periodontitis due to circadian rhythm disruption. **a** H&E staining of periodontal tissues, the region enclosed by the yellow dotted line indicates areas of inflammatory tissue and cellular infiltration. Scale bar is 50 μm. (E, epithelium; A, alveolar bone; T, tooth; yellow line indicates epithelial basement membrane; black line indicates alveolar bone crest); **b** TRAP staining of periodontal tissues, the number of TRAP-positive osteoclasts (indicated by red arrows). Scale bar is 50 μm; **c**, **d** IHC staining of IL-10, and TNF-α in periodontal tissues. Scale bars are 50 μm; **e**, **f**, **g**, **h** Gingival tissue was collected from mice treated with Gala-SR, SR1078, and DMSO in Jet lag. Real-time qPCR analyses were performed to determine mRNA expression of *Tnf-α, Il-6, Il-1β,* and *Il-10* in periodontal tissues of mice (*n* = 4, *P < 0.05, **P < 0.01, ***P < 0.001, ****P < 0.000 1)

Histological examination through hematoxylin-eosin (HE) staining revealed significant tissue alterations induced by ligature placement, including pronounced inflammatory cell infiltration and epithelial disruption. Comparative analysis demonstrated that both Gala-SR and SR1078 treatment groups exhibited attenuated periodontal tissue destruction compared to the DMSO-treated controls. Specifically, these therapeutic groups maintained superior epithelial integrity, preserved alveolar bone height, and showed reduced subepithelial inflammatory cell accumulation (Fig. 7a). Osteoclastic activity, assessed through tartrate-resistant acid phosphatase (TRAP) staining, was significantly elevated in the DMSO-treated periodontitis group compared to healthy control mice. Notably, both Gala-SR and SR1078 treatment groups exhibited a marked reduction in TRAP-positive cell numbers relative to the DMSO-treated periodontitis controls, indicating suppressed osteoclastic activity (Fig. 7b) and further substantiated the superior efficacy of Gala-SR in suppressing osteoclast activities and inflammation within the periodontal tissue in Jet Lag mice. The expression of IL-10, a crucial repair-related cytokine, was significantly downregulated in both DMSO-LD and DMSO-Jet Lag groups. In contrast, therapeutic intervention with Gala-SR and SR1078 markedly increased IL-10-positive cell populations (Fig. 7c, h). These findings suggest that RORα activation facilitates alveolar bone regeneration under both circadian rhythm-disrupted and normal periodontal conditions. Previous studies have established that circadian rhythm disruption potently activates the NF-κB signaling cascade. Consistent with this mechanism, our experimental data demonstrate that Gala-SR and SR1078 treatments significantly downregulated key NF-κB pathway mediators, including *Tnf-α* and *Il-6* (Fig. 7d–f), indicating effective suppression of NF-κB-mediated inflammatory signaling to alleviate alveolar bone loss.

## Discussion

The increasing prevalence of high hydrophobicity and intrinsically low aqueous solubility among lead compounds, drug development candidates, and marketed pharmaceuticals presents significant challenges in pharmaceutical development.^34^ Poor aqueous solubility represents a critical risk factor for inadequate oral bioavailability, as drug molecules must typically exist in a dissolved state to facilitate absorption.^42^ Consequently, medicinal chemistry strategies during lead optimization frequently focus on modifying physicochemical properties, particularly solubility, to enhance drug developability. Notably, nearly all commercially available synthetic small-molecule modulators targeting circadian rhythms exhibit high hydrophobicity and intrinsically low solubility.^42,43^ In this study, we utilized monosaccharide^44^ and disaccharide^45^ conjugation as a prodrug strategy to structurally modify SR1078, the sole commercially available RORα agonist, establishing a paradigm for optimizing circadian rhythm-modulating compounds. Through comprehensive evaluation of solubility, biocompatibility, and target engagement efficacy among three derivatives, such as representative monosaccharide conjugates (galactose and mannose) and a disaccharide conjugate (maltose), Gala-SR demonstrated superior overall performance.

Pharmacokinetic profiling in mice via intraperitoneal injection revealed that Gala-SR exhibited significantly prolonged systemic exposure, with plasma concentrations at the 8 hour timepoint substantially exceeding those of SR1078. This observation suggests that galactose modification markedly enhances the pharmacokinetic profile of SR1078. However, the bioavailability of Gala-SR and SR1078 was comparable, which may be attributed to two primary factors. One reason is that, as a lipophilic small molecule, S1078 is susceptible to adsorption by peritoneal adipose tissue, resulting in delayed release into systemic circulation in IP administration, which potentially lead to variability in AUC calculations and subsequent overestimation of bioavailability. The additional reason may involve the relatively small sample size in this study may have limited statistical power, necessitating larger cohorts in future study.

The conversion of SR1078 to Gala-SR was achieved through a hydrolysable acetal linker, which undergoes cleavage by endogenous galactosidases to release the parent drug (SR1078) and the sugar moiety. The enzymatic hydrolysis of Gala-SR by galactosidase to release SR1078 was found to be crucial for efficient activation of RORα. This enzyme-dependent activation was confirmed by the significant reduction in RORα-targeting efficacy upon co-treatment with a galactosidase inhibitor. To further elucidate the binding interactions, we performed molecular docking and molecular dynamics (MD) simulations to compare the binding modes of SR1078 and Gala-SR with RORα. Lower root-mean-square deviation (RMSD) values indicated reduced deviations from the reference structure, reflecting a closer approximation to a stable conformation. Similarly, smaller radius of gyration (Rg) values implied a more compact molecular architecture, typically associated with greater energetic stability.^46^ These computational analyses collectively demonstrate that SR1078 forms a more stable binding state with RORα compared to Gala-SR, highlighting the critical role of the hydrolysable acetal linker in determining the efficacy of Gala-SR-mediated targeted activation of RORα.

Further in vitro and in vivo investigations demonstrated that RORα activation by Gala-SR significantly enhanced the amplitude and expression of BMAL1 without altering the period or phase. Notably, Gala-SR exhibited dose-dependent efficacy, whereas SR1078 lacked this characteristic, likely due to its poor aqueous solubility, which limits its effective concentration in the culture medium. In contrast, the significantly improved aqueous solubility of Gala-SR enhanced its cellular utilization efficiency, facilitating its targeted activation of RORα. Our findings in human periodontal ligament stem cells (hPDLSCs) further revealed that Gala-SR more potently activated RORα function and upregulated *BMAL1* expression compared to SR1078. These results provide compelling evidence supporting the potential therapeutic application of Gala-SR in periodontal tissues. Comparative assessment of in vivo biocompatibility showed that low-dose SR1078 administration did not induce significant adverse effects in mice. However, dose-dependent toxicity became evident with increasing SR1078 dosage. These findings suggest that Gala-SR exhibits favorable biocompatibility, effectively enhancing circadian rhythm amplitude in a safe and controlled manner in vivo.

We noticed that the loss of alveolar bone, infiltration of immune cells, and number of osteoclasts in periodontal tissue all showed a more benign status in the Jet Lag mice treated with two modulators than that in mice of the DMSO-LD group. It suggests an anti-inflammatory effect alongside the restoration of circadian rhythm, that Gala-SR and SR1078 may exert by targeted activation of RORs. In a previous study, we found a deficient level of local RORα in the refractory diabetic bone defect. Restoring the level of RORα promoted diabetic bone defect repair by positively regulating the transcription of *ccl3* and *IL-6* to activate macrophage-induced migration and proliferation of BMSCs in the early stage of bone repair.^47^ Together with our findings herein, it can be surmised that RORα has a dual function in both the circadian and inflammatory systems, which enables its targeted modulators, such as Gala-SR, to exert potential therapeutic effects in diseases caused by or accompanied by rhythm disorders, particularly the chronic inflammatory diseases. Considering that previous studies have identified clock-amplitude-enhancing molecules (CEMs) to ameliorate circadian rhythm disorders.^48^ Apparently, the patterns of Gala-SR affecting the circadian genes and inflammatory activities in periodontal tissue affirmed itself a CEM that targeted activates RORα to restore the circadian rhythm of periodontal tissue, thereby alleviating periodontitis and alveolar bone loss, thus demonstrating great promise in the therapeutic interventions of circadian rhythm disorder and related inflammatory diseases. Notably, after intraperitoneal injection of Gala-SR to the mice with disordered circadian rhythm, the expression of circadian genes in both the liver (Fig. 5e) and periodontal tissue (Fig. 6c) was restored. These findings indicate that Gala-SR is effective systemically, that may also of great promise for the therapy of systemic inflammatory diseases related to RORα abnormalities, such as primary Sjogren’s syndrome,^49^ diabetes,^50^ and systemic lupus erythematosus.^51^

Monosaccharides, such as galactose and glucose, are polar and non-toxic nutrients that are highly attractive in prodrug modification strategies.^27^ While improving the aqueous solubility, monosaccharides can further utilize SGLT1 and GLUT2 in the small intestine to help transport drug molecules into the bloodstream, thereby offering a favorable oral bioavailability.^30^ The acetal linker utilized in this study to conjugate galactose onto SR1078 is not only sensitive to β-hydrolase, but also reported to be acid stable. This characteristic renders Gala-SR and molecules sharing similar prodrug design feature potentially resistant to gastric acid digestion, further facilitating their uptake by the small intestine. Given that RORα is widely distributed throughout the body, oral administration of RORα modulators undoubtedly provides a safe and convenient way to regulate circadian rhythms systemically. In the future, based on Gala-SR, we will further investigate the oral administration characteristics of monosaccharide modified modulators targeting RORα, in order to bring new strategies for the treatment of circadian rhythm disorders and related diseases.

## Materials and methods

### Cell culture

HEK 293 T cells, HepG2, hPDLCs and human U2OS derived cell lines were cultured at 37 °C with a 5% CO_2_ atmosphere in Dulbecco’s Modified Eagle’s Medium (HyClone) supplemented with 10% fetal bovine serum (Gibco) and 1% Penicillin Streptomycin mixture (MCE).

### Transient transfection

HEK293T cells were transfected with Lipofectamine 8000 (ThermoFisher) strictly following the manufacturer’s instructions. Cotransfected HEK293T cells with RORα or RORγ plasmid and a reporter consisting of the *G6Pase* or *FGF21* promoters upstream of a luciferase reporter gene. 500 ng plasmid DNA was transfected into each well of 24 well-plate 24 h after the transfection, DMSO or Gala-SR and SR1078 (5 µmol/L) was added and incubated for 24 h. The Lumicycle instrument detects luminescence and records the numerical value.

### Real-time bioluminescence monitoring

Two clonal circadian reporter cell lines, *Bmal1-dLuc* and *Per2-dLuc*, were established and used in this study.^36^ These two cell lines display robust cellular rhythmicity with near anti-phasic oscillation of bioluminescence expression. The lentivirus preparation, transduction of U2OS cells and selection of the knockout candidates with puromycin (at 0.5 mg/mL concentration) were performed using a previously published protoco.^37^ LumiCycle (Actimetrics, Inc., Evanston, IL) detect for U2OS cells stably expressing Bmal1-dLuc and Per2-dLuc cells were seeded on an opaque 24-well plate. When the cells are full, which were treated with dexamethasone (DEX) (100 nmol/L final) for 2 h for resetting, 2 h later, the media of cells were replaced with bioluminescence recording media having DMEM culture medium (Gibco 21063029, 500 mL), 70 mL 12% FBS. Luciferin (0.1 mmol/L final) was added fresh to the bioluminescence media. Molecules at determined concentrations were supplied to the recording media (final DMSO concentration is 0.1%). To prevent evaporation and gas exchange, and hence to maintain cell homeostasis, optically clear film was used to seal plates.

### Solubility test

Prepare test compounds in DMSO at 10 mmol/L, prepare test compounds in DMSO at 10 mmol/L, put a PTFE encapsulated stainless steel stick stirrer into each vial, then seal vials using a molded PTDE/SIL silicone plug, transfer the solubility sample plate to the Thermomixer Comfort plate shaker, 1 100 r/min, 25 °C for 2 h, remove the stir sticks using a big magnet after 2 h’ incubation, transfer the solubility plate sample into the filter plate using Vacuum Manifold, transfer 10 µL the filtered samples and 10 µL DMSO into 980 µL methanol, further diluted 10- fold in methanol:water (1:1) as filtered samples for LC-MS/MS analysis, calculate the solubility using Microsoft Excel.

$${\rm{Solubility}}({\rm{\mu mol/L}})=\frac{{{Area}}_{{\rm{filtered}}}\times {{DF}}_{{\rm{filtered}}}}{{{Area}}_{{\rm{std}}}\times {{DF}}_{{\rm{std}}}}\times [{\rm{std}}]\times {{DF}}_{{\rm{std}}}$$

DF: the dilution factor.

### CCK-8 assay

U2OS cells were seeded at a density of 10 000 cells per well in a 96-well plate, with 100 μL of culture medium added to each well. The cells were incubated at 37 °C in a 5% CO_2_ atmosphere for 24 h or until they reached the logarithmic growth phase. Following this, the cells were treated with SR1078, Gala-SR, Malt-SR, and Mann-SR and incubated for 24, 48, and 72 h. 10 μL of CCK-8 reagent was added to each well, and the plate was returned to the incubator for an additional 1–4 h. Absorbance at 450 nm was then measured using a microplate reader. The average absorbance for each well was calculated, and relative cell viability was determined by comparing the absorbance values to those of the control group.

### Total RNA extraction

Cells were seeded into 6-well plates, treated with the drug for 24 h, and total RNA samples were extracted using TRIzol reagent (Vazyme, China). The extraction of total RNA was performed according to the following steps: After removal of the supernatant, the cells were thoroughly washed three times with phosphate-buffered saline (PBS). Subsequently, the cells were lysed by the application of 1 mL TRIzol reagent. The resulting samples were harvested into frozen 2 mL Eppendorf microcentrifuge tubes and incubated at room temperature for 5 min. A volume of 0.2 mL of chloroform was added to the microcentrifuge tubes to ensure homogenization of the samples, which were then vortexed and incubated at room temperature for another 10 min. The samples were then centrifuged at a speed of 12 000 × *g* in a microcentrifuge at 4 °C for 15 min. The upper aqueous phase was subsequently collected and transferred to a new 2 mL microcentrifuge tube and mixed with an equal volume of isopropanol. The mixture was then centrifuged at a speed of 12 000 × *g* in a table microcentrifuge at 4 °C for 10 min. The resulting precipitates were washed once with 1 mL of 75% ethanol and once with 100% ethanol, followed by centrifugation at a speed of 7 500 × *g* in a table microcentrifuge for 5 min. Finally, the pellet was dissolved in RNase-free DEPC-treated water.

### Reverse transcription PCR and real-time qPCR

RNA from liver, periodontal tissues and cell samples was prepared as previously described. Total RNA (1 ug) was synthesized into cDNA using AceQ qPCR SYBR Green Master Mix (Vazyme). mRNA expression levels were normalized to GAPDH. Relative expression levels and enrichment contents were calculated using the comparative Ct Method. Primer information is detailed in Data file S1.

### Immunofluorescent staining

U2OS cells were seeded on coverslips and treated with drugs for 24 h. After treatment, the cells were washed 3 times with PBS and fixed with 4% paraformaldehyde at room temperature for 15 min. The cells were then permeabilized with 0.1% Triton X-100 (diluted in PBS) and blocked with 5% BSA (diluted in PBS) at room temperature for 30 min. Primary antibodies were incubated overnight at 4 °C. The next day, the cells were washed 3 times with PBST and incubated with Cy3-conjugated Affinipure Goat Anti-Rabbit IgG(H + L) secondary antibody (1:200) at room temperature for one hour, with DAPI added to stain the nuclei. After washing 3 times with PBST, images were captured using a laser confocal microscope (Nikon A1-Si).

### Real-time bioluminescence recording of mice

PER2::LUC mice at ages of 2–3 months-old were housed in 12:12 light-dark conditions for two weeks before the experiments.^33^ Gala-SR and SR1078 (10 mg/kg) were dissolved in DMSO and I.P.injected into mouse at ZT0. After 1 h, D-luciferin was I.P. injected into mouse for another 0.5 h. Mice were then imaged on IVIS 673 Lumina III system and analyzed with live imaging software.

### Pharmacokinetic studies

A single 10 mg/kg i.p. dosage of Gala-SR and SR1078 were administered to 8-week-old male C57BL/6 J mice (*n* = 3 at each time point). After administering Gala-SR and SR1078, blood samples were taken 0, 0.5, 1, 2, 4, 8, 12 and 24 h later by heart puncture while under isoflurane anesthesia. Heparinized tubes were used to extract plasma, which was then centrifuged and kept at −80 °C until analysis.

### Molecular docking

Perform molecular docking of Gala-SR and SR1078 with RORα using Autodock Vina, ensuring the quality and file format suitability (such as PDB or Mol2) of SR1078’s structure for docking. Convert the structures of SR1078 and Gala-SR into a suitable format for docking using Openbabel 2.4.1. Preprocess the protein by obtaining the crystal structure of RORα and utilizing Autodock Tools4. Configure the docking parameters with Autodock Vina v1.2, followed by visualization of the docking results using Autodock Tools4 and PyMol 2.5.0.

### Molecular dynamics simulation

Molecular dynamics simulations of the protein-ligand complex were performed using Amber20. To accurately describe the different molecular components within the system, the ff14SB force field was selected for the protein, while the gaff2 force field was used for the small molecule, with force field parameters generated by Antechamber. The simulation system was solvated using the TIP3P water model, and an appropriate amount of ions was added to achieve system neutrality. During the preparation phase, a two-step energy minimization was conducted: first, a spatial constraint of 10 kcal/mol·Å² was applied to the heavy atoms of the solute, followed by 2 500 steps of energy minimization; then, all constraints were removed, and 5 000 steps of unconstrained minimization were performed on the entire system. During the heating process, the system was gradually heated from 0 to 300 K using Langevin dynamics, with a damping coefficient set to 2.0, and a constraint of 5 kcal/mol·Å² was applied to non-water and non-hydrogen atoms. This phase lasted for 25 000 steps, with a time step of 2 fs, to ensure the system gradually reached the target temperature. Subsequently, during the equilibration phase, three sub-steps were performed, gradually reducing the constraint force on the backbone atoms under constant pressure (NPT) conditions. First, a constraint of 5 kcal/mol·Å² was applied for 100 000 steps; then, the constraint was reduced to 1 kcal/mol·Å² for another 100 000 steps; finally, a slight constraint of 0.1 kcal/mol·Å² was applied for an additional 100 000 steps, allowing the system to gradually approach its natural state. During the production simulation phase, all constraints were removed, and the simulation continued under constant pressure conditions for 50 000 000 steps, reaching a total duration of 100 ns to ensure free evolution of the system. The simulation temperature was set to 300 K, and the pressure was maintained at 1 atm using the Berendsen barostat method. Trajectory data were saved every 10 ps. The cutoff distance for non-bonded interactions was set to 9.0 Å, with an integration time step of 2 fs, and the SHAKE algorithm was applied to constrain hydrogen bonds.

### Mouse locomotor assay

WT C57BL/6 J mice at the age of 2 months old were entrained under a 12-h /12-h dark cycle at 24 °C for 2 weeks.^36^ One week after mouse clock synchronization to the ambient light/dark cycle, SR1078, Gala-SR or dimethyl sulfoxide (DMSO) was dissolved in saline and intraperitoneally injected (10 mg/kg) into mice 1 h before light off (ZT11). Light/dark cycles were subsequently phase advanced or delayed by 8 h. SR1078, Gala-SR or DMSO was injected again 24 h later. After 2 weeks of continuous recording, locomotor activity was analyzed with ClockLab software (Actimetrics) following the procedures described previously34.

### Micro-CT

Alveolar bones were harvested from mice and fixed after removal of soft tissues. The bones were then scanned at a resolution of 9 μm using Micro-CT (SkyScan 1176, Bruker). Images were reconstructed and quantified using InstaRecon/NRecon Research Workplace software. Metrics such as bone volume/total volume, trabecular thickness, bone surface area/bone volume, trabecular number, trabecular separation, and buccal-lingual alveolar bone resorption were used to assess differences among the mice.

### Establishment of the periodontitis animal model

Male C57BL/6 mice,^40^ 2 months old, were housed under disrupted conditions for 4 weeks. From the third week, they were intraperitoneally injected with Gala-SR, SR1078, or DMSO for 3 weeks. In the fifth week, general anesthesia was administered via intraperitoneal injection of pentobarbital sodium (40 mg/kg), and a ligature was placed around the cervix of the maxillary second molar to establish the murine periodontitis model. Collect gingival tissue and liver tissue every 6 h continuously for a duration of 48 h.

### Statistical analysis

In all experiments, error bars represent the standard deviation (SD) unless otherwise specified. Statistical significance was determined using unpaired two-sided Student’s *t*-tests for comparisons between two groups with normally distributed data. One-way analysis of variance (ANOVA) was employed for analyses involving more than two groups. A *P*-value less than 0.05 was considered statistically significant. Each group consisted of at least three samples, unless explicitly stated.

## Supplementary information

Supporting material-BONERES-04018R--marker

Supporting material-BONERES-04018R

## Data Availability

The entirety of the data pertaining to this study can be found within the confines of both the paper and the Supplementary Materials. The authors can be contacted for additional data related to this paper. The material methods described in the text are included as supplementary information in the Materials Supplementary Materials section.
