# Supplementary material for: Galactose-modified small molecule modulator targets RORα to enhance circadian rhythm and alleviate periodontitis-associated alveolar bone loss
Source: Bone Res. 2025 Oct 30;13:91. doi: 10.1038/s41413-025-00445-w (PMC12575728; doi:10.1038/s41413-025-00445-w)
Supplement: Supplementary file 1 — Supporting material-BONERES-04018R--marker [file 41413_2025_445_MOESM1_ESM.pdf]

Galactose-modified small molecule modulator targets RORα to enhance circadian rhythm and alleviate periodontitis-associated alveolar bone loss

Guangxia Feng†1,2,3, Zhiwen Liao†4, Yifan Wang†2,3*, Qingming Tang1,2,3, Nayun Li1,2,3, Cheng Li1, Yuqing Liu1,2,3, Renlong Liu1,2, Mingjian Cui 4, Wenjie Fan1,2,3, Ying Yin1,2,3, Lingkui Meng4, Jing Zeng4, Zetao Chen5, Guanzheng Luo6, Peng Xiang6, Qian Wan4 *, Lili Chen1,2,3,5*

Aff1 Department of Stomatology, Union Hospital, Tongji Medical College, Huazhong University of Science and Technology, Wuhan, Hubei, China

Aff2 School of Stomatology, Tongji Medical College, Huazhong University of Science and Technology, Wuhan, Hubei, China

Aff3 Hubei Province Key Laboratory of Oral and Maxillofacial Development and Regeneration, Wuhan, Hubei, China

Aff4 Hubei Key Laboratory of Natural Medicinal Chemistry and Resource Evaluation, School of Pharmacy, Huazhong University of Science and Technology, 13 Hangkong Road, Wuhan, Hubei, China

Aff5 Hospital of Stomatology, Guanghua School of Stomatology, Sun Yat-sen University, Guangdong Research Center for Dental and Cranial Rehabilitation and Material Engineering, Guangzhou, China

Aff6 School of Life Sciences, Sun Yat-sen University, Guangzhou, China

† These authors contributed equally: Guangxia Feng, Zhiwen Liao, Yifan Wang

* Correspondence: Lili Chen (E-mail:chenlili1030@hust.edu.cn, Tel: +86-27-85726949 Fax: +86-27-85726949)

Qian Wan (E-mail: wanqian@whu.edu.cn)

Yifan Wang (E-mail: yifan24_wang@hust.edu.cn)

The supplementary material file includes:

Figure S1. ^{1}H NMR (400 MHz, DMSO-*d6*) spectrum of **SR1078**

Figure S2. ^{1}H NMR (600 MHz, $CDCl_3$) spectrum of **2**

Figure S3. ^{13}C NMR (150 MHz, $CDCl_3$) spectrum of **2**

Figure S4. ^{19}F NMR (565MHz, $CDCl_3$) spectrum of **2**

Figure S5. ^{1}H NMR (600 MHz, CD_3OD) spectrum of **Gala-SR**

Figure S6. ^{13}C NMR (150 MHz, CD_3OD) spectrum of **Gala-SR**

Figure S7. ^{19}F NMR (565MHz, CD_3OD) spectrum of **Gala-SR**

Figure S8. ^{1}H NMR (600 MHz, $CDCl_3$) spectrum of **4**

Figure S9. ^{13}C NMR (150 MHz, $CDCl_3$) spectrum of **4**

Figure S10. ^{19}F NMR (565 MHz, $CDCl_3$) spectrum of **4**

Figure S11. ^{1}H NMR (600 MHz, CD_3OD) spectrum of **Mann-SR**

Figure S12. ^{13}C NMR (150 MHz, CD_3OD) spectrum of **Mann-SR**

Figure S13. ^{19}F NMR (565 MHz, CD_3OD) spectrum of **Mann-SR**

Figure S14. ^{1}H NMR (400 MHz, $CDCl_3$) spectrum of **7**

Figure S15. ^{13}C NMR (150 MHz, $CDCl_3$) spectrum of **7**

Figure S16. ^{1}H-^{1}HCOSY NMR (400 MHz, $CDCl_3$) spectrum of **7**

Figure S17. ^{1}H NMR (600 MHz, $CDCl_3$) spectrum of **8**

Figure S18. ^{13}C NMR (150 MHz, $CDCl_3$) spectrum of **8**

Figure S19. ^{19}F NMR (565 MHz, $CDCl_3$) spectrum of **8**

Figure S20. ^{1}H-^{1}HCOSY NMR (600 MHz, $CDCl_3$) spectrum of **8**

Figure S21. ^{1}H NMR (600 MHz, CD_3OD) spectrum of **Malt-SR**

Figure S22. ^{13}C NMR (150 MHz, CD_3OD) spectrum of **Malt-SR**

Figure S23. ^{19}F NMR (565 MHz, CD_3OD) spectrum of **Malt-SR**

Figure S24. ^{1}H-^{1}HCOSY NMR (600 MHz, CD_3OD) spectrum of **Malt-SR**

Figure S25. Live/dead staining of U2OS cells treated with different saccharide-modified SR1078

Figure S26.Galactose modification improves biocompatibility *in vivo*

Figure S27.Galactose modification dose-dependently activates RORs-directed transcription.

Figure S28. Gala-SR improved pharmacokinetic characteristics

Figure S29.Exploring the impact of acetal linker design on RORγ-targeted regulatory function

Figure S30. Gala-SR enhances circadian rhythm *in vitro*

Figure S31. ^{1}H NMR (400 MHz, DMSO-*d*6) spectrum of **9**

Figure S32. ^{13}C NMR (150 MHz, DMSO-*d*6) spectrum of **9**

Figure S33. ^{19}F NMR (565 MHz, DMSO-*d*6) spectrum of **9**

Figure S34. Exploration of the inhibitory effect of compound 9 on the targeting function of RORs

Figure S35.Impact of Gala-SR on circadian clock gene expression in periodontal ligament stem cells.

Figure S36.Toxicity and safety evaluation *in vivo*

Synthetic Partial Materials

1. General Comments

All reactions were monitored by thin-layer chromatography over silica-gel-coated TLC plates (Yantai Chemical Industry Research Institute). The spots on TLC were visualized by warming 10% H_2SO_4 (10% H_2SO_4 in ethanol) sprayed plates on a hot plate. Column chromategraphy was performed using silica gel (Qingdao Marine Chemical Inc., China) Sephadex LH-20 (GE Healthcare Bio-Sciences AB, Sweden). NMR spectra were recorded on a Bruker AM-400 spectrometer (400 MHz) and Bruker Ascend TM-600 spectrometer (600 MHz), and the ^{1}H NMR and ^{13}C NMR chemical shifts were referenced to the solvent or solvent impurity peaks for $CDCl_3$ at δ_H 7.24 and δ_C 77.23, for DMSO-*d*6 at δ_H 2.50 and δ_C 39.52, for CD_3OD at δ_H 3.31 and δ_C 49.00. All reaction were heated by metal sand bath (WATTCAS, Z100500, http://www.xinweier.com/Product/Product_Info.aspx?ProductID=4140). High resolution mass spectra (HRMS) were recorded on a Bruker micrOTOF II spectrometer using electrospray ionization (ESI). LC/MSD Trap 2D Liquid Chromatography-Ion Trap Mass Spectrometry (LC-MS) were recorded on a thermos scientific Q Exactive HR MS using electrospray ionization (ESI). Optical rotations were measured at 25 °C with a Rudolph Autopol IV automatic polarimeter using a quartz cell with 1 mL capacity and a 1 dm path length. Concentrations (*c*) are given in g/100 mL.

2. Materials

Solvents for reactions were dried on an Innovative Technologies Pure Solv400 solvent purifier. Molecular sieves (4Å, powder < 50 μm) for reactions were flame dried immediately before use. All other chemicals were purchased from Adamas and used without further purification.

3. Preparation of SR1078, Gala-SR, Mann-SR, Malt-SR and compound 9

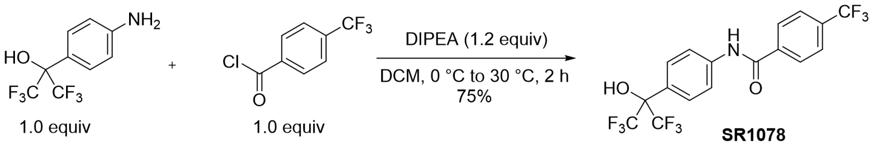

N-(4-(1,1,1,3,3,3-hexafluoro-2-hydroxypropan-2-yl)phenyl)-4-(trifluoromethyl)benzamide (**SR1078**)

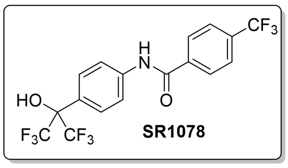

55.2-(4-Aminophenyl)-1,1,1,3,3,3-hexafluoropropan-2-ol (2.0 g, 7.72 mmol) was dissolved in DCM under argon atmosphere. 4-(trifluoromethyl)benzoyl chloride (1.2 mL, 7.72 mmol) and DIPEA (1.6 mL, 9.26 mmol) was added to the above solution at 0 ºC. After stirring 10 min at 0 ºC, the reaction mixture was warmed up to 30 °C and stirred for 2 h and extracted with EtOAc. The organic phase was washed with saturated brine, dried over anhydrous Na_2SO_4, concentrated *in vacuo*. The crude product was recrystallized with EtOAc/petroleum ether and filtration, the **SR1078** (2.5 g, 75%) was obtained as a white solid. R*f* = 0.33 (petroleum ether-EtOAc 4:1). ^{1}H NMR (400 MHz, DMSO-*d*6) δ 10.67 (s, 1H), 8.66 (s, 1H), 8.16 (d, *J* = 8.0 Hz, 2H), 7.93 (d, *J* = 8.8 Hz, 4H), 7.69 (d, *J* = 8.4 Hz, 2H). Analytical data for **SR1078** were essentially the same as reported in the literature[1].

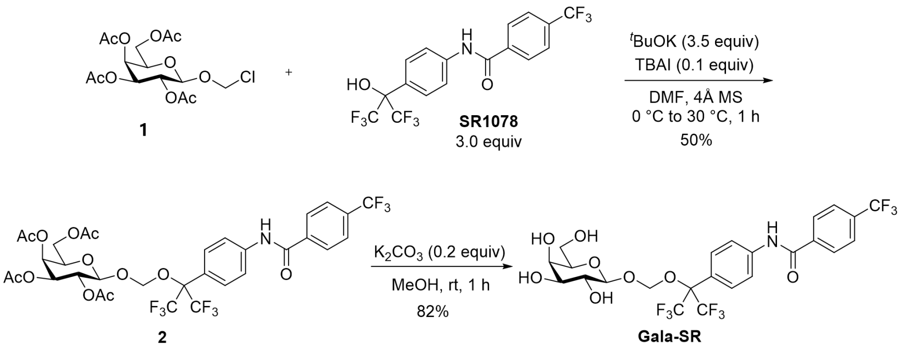

N-(4-(1,1,1,3,3,3-hexafluoro-2-(2,3,4,6-tetra-*O*-acetyl-*β*-D-galactopyranosyl)-2-yl)phenyl)-4-(trifl uoromethyl)benzamide (**2**)

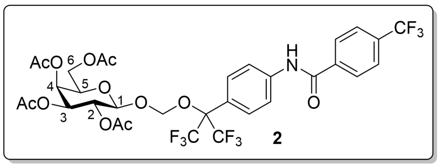

SR1078 (235.5 mg, 0.53 mmol), ^{t}BuOK (69.2 mg, 0.62 mmol), TBAI (6.5 mg, 0.017 mmol) was dissolved in dry DMF (0.8 mL) in the present of 4Å MS (100 mg/mL) under argon atmosphere. After stirring 10 min at 0 ºC, **1**[2] (70 mg, 0.17 mmol) was dissolved in DMF (0.8 mL) and then added to above solution. Then the reaction mixture warmed up to 30 °C and stirred for 1 h and extracted with EtOAc. The organic phase was washed with saturated brine, dried over anhydrous Na_2SO_4, concentrated *in vacuo*, and purified by flash column

chromatography on silica gel to give **2** (70 mg, 50%) as a colorless syrup. $[\alpha]_D^{25}$ -111.9 (*c*, 1.0 in $CHCl_3$). R*f* = 0.30 (petroleum ether-EtOAc 2:1). ^1H NMR (600 MHz, $CDCl_3$) δ 8.01 (s, 1H, -NH-), 7.98 (d, *J* = 7.8 Hz, 2H), 7.77 (m, 4H), 7.59 (d, *J* = 9.0 Hz, 2H), 5.41 (d, *J* = 3.0 Hz, 1H, **H-4**), 5.25 (dd, *J* = 10.4, 7.8 Hz, 1H, **H-2**), 5.18 (d, *J* = 6.6 Hz, 1H, -OCH_2-), 5.08 (dd, *J* = 10.4, 3.0 Hz, 1H, **H-3**), 4.90 (d, *J* = 7.8 Hz, 1H, **H-1**), 4.86 (d, *J* = 6.6 Hz, 1H, -OCH_2-), 4.17 (dd, *J* = 11.4, 6.6 Hz, 1H, **H-6a**), 4.10 (dd, *J* = 11.4, 6.6 Hz, 1H, **H-6b**), 3.97 (t, *J* = 6.6 Hz, 1H, **H-5**), 2.13 (s, 3H, -OAc), 2.07 (s, 3H, -OAc), 2.04 (s, 3H, -OAc), 1.98 (s, 3H, -OAc). ^{13}C NMR (150 MHz, $CDCl_3$) δ 170.7, 170.4, 170.3, 169.9, 164.8, 139.9, 137.9, 134.2 (q, *J* = 32.7 Hz), 129.5, 127.8, 126.2 (q, *J* = 3.6 Hz), 124.7, 123.7 (q, *J* = 270.9 Hz), 120.4, 98.0, 88.6, 71.2, 71.0, 68.6, 67.1, 61.3, 20.9, 20.8, 20.8, 20.8. ^{19}F NMR (565 MHz, $CDCl_3$) δ -63.03 (s), -71.14 (q, *J* = 10.0 Hz), -72.10 (q, *J* = 10.0 Hz). HRMS (ESI-TOF) m/z: $[M+Na]^+$ calc. for $C_{32}H_{30}F_9NO_{12}Na$ 814.1517, found: 814.1557.

N-(4-(1,1,1,3,3,3-hexafluoro-2-(*β*-D-galactopyranosyl)-2-yl)phenyl)-4-(trifluoromethyl)benzamide (**Gala-SR**)

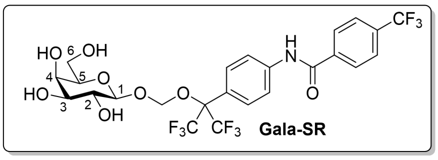

61.To a stirred solution of **2** (70 mg, 0.088 mmol) in MeOH (0.9 mL) was added K_2CO_3 (2.4 mg, 0.018 mmol) at room temperature. The mixture was stirred for 1 h and directly purified by column chromatography on Sephadex LH-20 to afford compound **Gala-SR** (45 mg, 82%) as a white crystal. R*f* = 0.30 (DCM-MeOH 8:1). m.p. 94.3-96.8 °C. $[\alpha]_D^{25}$ -104.7 (*c*, 0.53 in CH_3OH). ^1H NMR (600 MHz, CD_3OD) δ 8.12 (d, *J* = 8.4 Hz, 2H), 7.92 (d, *J* = 9.0 Hz, 2H), 7.84 (d, *J* = 8.4 Hz, 2H), 7.75 (d, *J* = 8.4 Hz, 2H), 5.31 (d, *J* = 6.0 Hz, 1H, -OCH_2-), 4.95 (d, *J* = 6.0 Hz, 1H, -OCH_2-), 4.64 (d, *J* = 7.2 Hz, 1H, **H-1**), 3.87 (d, *J* = 3.0 Hz, 1H, **H-4**), 3.77 – 3.71 (m, 2H, **H-2, H-3**), 3.62 – 3.57 (m, 2H, **H-5, H-6a**), 3.54 (dd, *J* = 9.6, 3.0 Hz, 1H, **H-6b**). ^{13}C NMR (150 MHz, CD_3OD) δ 167.5, 142.0, 139.8, 134.4 (q, *J* = 32.1 Hz), 130.4, 129.5, 126.6 (q, *J* = 3.3 Hz), 125.8 (q, *J* = 269.7 Hz), 124.5, 121.9, 102.2, 90.3, 77.0, 74.8, 72.2, 70.1, 62.4. ^{19}F NMR (565 MHz, CD_3OD) δ -64.47 (s), -72.65 (q, *J* = 10.0 Hz), -72.93 (q, *J* = 10.0 Hz). HRMS (ESI-TOF) m/z: $[M+Na]^+$ calc. for $C_{24}H_{22}F_9NO_8Na$ 646.1094, found: 646.1095.

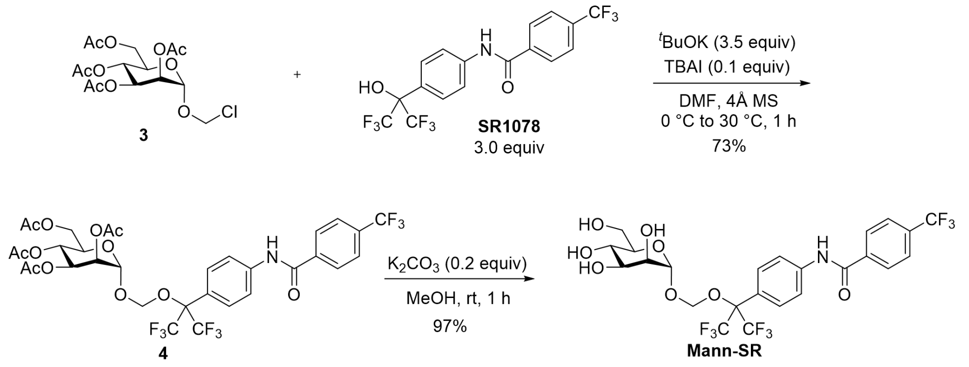

N-(4-(1,1,1,3,3,3-hexafluoro-2-(2,3,4,6-tetra-*O*-acetyl-*α*-D-mannopyranosyl)-2-yl)phenyl)-4-(trifluoromethyl)benzamide (**4**)

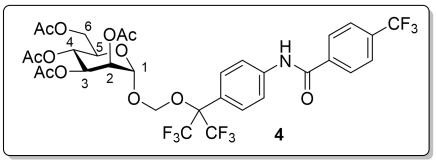

66.**SR1078** (337 mg, 0.76 mmol), ^{t}BuOK (99 mg, 0.88 mmol), TBAI (9 mg, 0.025 mmol) was dissolved in dry DMF (1.2 mL) in the present of 4Å MS (100 mg/mL) under argon atmosphere. After stirring 10 min at 0 ºC, **3**[2] (100 mg, 0.25 mmol) was dissolved in DMF (1.2 mL) and then added to above solution. Then the reaction mixture was warmed up to 30 °C and stirred for 1 h and extracted with EtOAc. The organic phase was washed with saturated brine, dried over anhydrous Na_2SO_4, concentrated *in vacuo*, and purified by flash column chromatography on silica gel to give **4** (144 mg, 73%) as a syrup. R*f* = 0.45 (petroleum ether-EtOAc 2:1). $[\alpha]_D^{25}$ +136.1 (*c*, 0.80 in $CHCl_3$). ^1H NMR (400 MHz, $CDCl_3$) δ 8.06 (brs, 1H), 7.99 (d, *J* = 8.4 Hz, 2H), 7.77 (m, 4H), 7.63 (d, *J* = 8.8 Hz, 2H), 5.35 – 5.27 (m, 3H, **H-2**, **H-3**, **H-4**), 5.19 (brs, 1H, **H-1**), 5.08 (d, *J* = 6.4 Hz, 1H, -OCH_2-), 4.90 (d, *J* = 6.0 Hz, 1H, -OCH_2-), 4.26 (dd, *J* = 12.4, 5.2 Hz, 1H, **H-6a**), 4.09 (dd, *J* = 12.4, 2.4 Hz, 1H, **H-6b**), 4.03 – 3.97 (m, 1H, **H-5**), 2.17 (s, 3H, -OAc), 2.07 (s, 3H, -OAc), 2.02 (s, 3H, -OAc), 1.99 (s, 3H, -OAc). ^{13}C NMR (150 MHz, $CDCl_3$) δ 170.9, 170.2, 170.1, 169.9, 164.8, 139.9, 137.9, 134.1 (q, *J* = 32.8 Hz), 129.6, 127.8, 126.2 (q, *J* = 3.5 Hz), 123.7, 120.5, 96.1, 88.8, 69.8, 69.2, 69.0, 66.0, 62.4, 21.1, 20.9. ^{19}F NMR (565 MHz, $CDCl_3$) δ -63.04 (s), -71.08 (q, *J* = 10.0 Hz), -71.78 (q, *J* = 10.1 Hz). HRMS (ESI-TOF) m/z: $[M+Na]^+$ calc. for $C_{32}H_{30}F_9NO_{12}$ Na 814.1517; found: 814.1499.

N-(4-(1,1,1,3,3,3-hexafluoro-2-(*α*-D-mannopyranosyl)-2-yl)phenyl)-4-(trifluoromethyl)benzamide (**Mann-SR**)

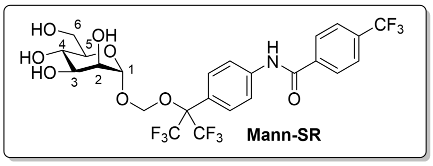

69.To a stirred solution of **4** (134 mg, 0.17 mmol) in

MeOH (1.7 mL) was added K_2CO_3 (5 mg, 0.034 mmol) at room temperature. The mixture was stirred for 1 h and directly purified by column chromatography on Sephadex LH-20 to afford compound **Mann-SR** (102 mg, 97%) as a white a crystal. R*f* = 0.31 (DCM-MeOH 8:1). m.p. 98.4-101.5 °C. $[\alpha]_D^{25}$ -232.3 (*c*, 0.70 in CH_3OH). ^1H NMR (400 MHz, CD_3OD) δ 8.13 (d, *J* = 8.4 Hz, 2H), 7.93 (d, *J* = 8.8 Hz, 2H), 7.85 (d, *J* = 8.0 Hz, 2H), 7.67 (d, *J* = 8.4 Hz, 2H), 5.27 (d, *J* = 6.8 Hz, 1H, -OCH_2-), 5.22 (d, *J* = 1.6 Hz, 1H, **H-1**), 4.88 (d, *J* = 7.2 Hz, 1H, -OCH_2-), 3.87 (m, 1H, **H-2**), 3.82 (dd, *J* = 11.6, 2.4 Hz, 1H, **H-6a**), 3.76 – 3.63 (m, 3H, **H-6b**, **H-3**, **H-4**), 3.54 – 3.48 (m, 1H, **H-5**). ^{13}C NMR (150 MHz, CD_3OD) δ 167.5, 142.2, 139.8, 134.4 (q, *J* = 32.3 Hz), 130.2, 129.5, 126.6 (q, *J* = 3.2 Hz), 124.4, 121.9, 99.2, 89.1, 75.6, 72.2, 71.8, 68.2, 62.7. ^{19}F NMR (565 MHz, CD_3OD) δ -64.48 (s), -71.36 (q, *J* = 10.3 Hz), -74.37 (q, *J* = 10.3 Hz). HRMS (ESI-TOF) m/z: $[M+Na]^+$ calc. for $C_{24}H_{22}F_9NO_8Na$ 646.1094; found: 646.1092.

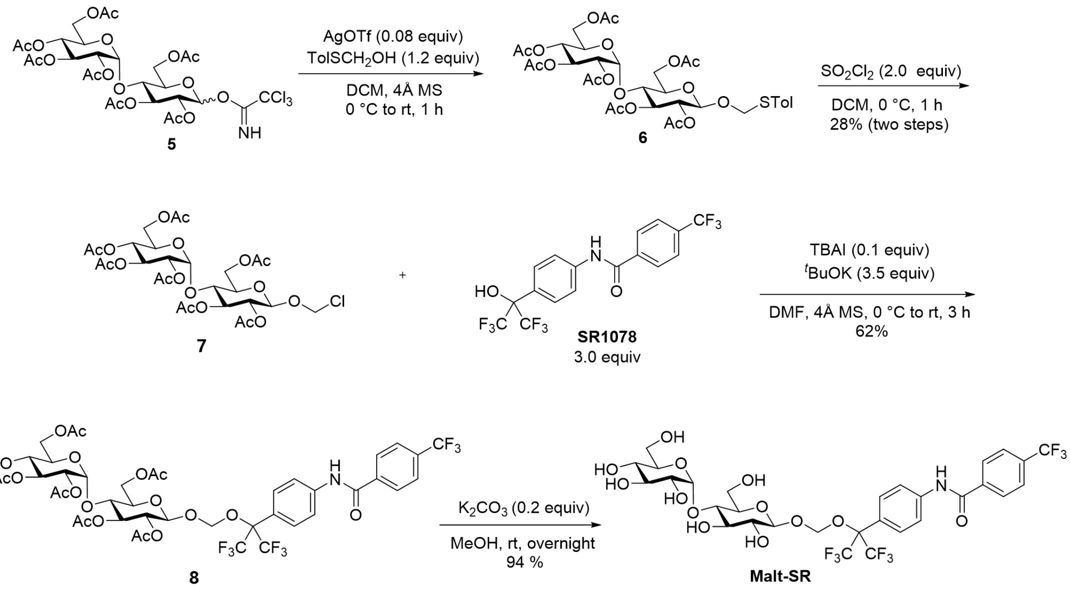

p-tolylthiomethyl-heptaacetyl-*β*-D-maltoside (**6**)

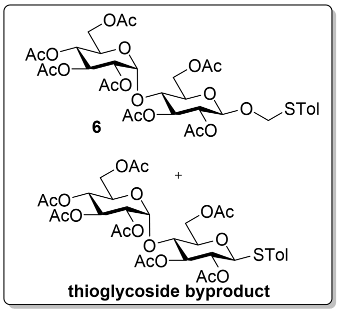

73.Compound **5**[3] (2.4 g, 3.07 mmol) and $TolSCH_2OH$ (568 mg, 3.69 mmol) was dissolved in dry DCM (30 mL) in the present of 4Å MS (100 mg/mL) under argon atmosphere. After stirring 10 min at 0 °C, AgOTf (63 mg, 0.25 mmol) was added to the above solution. Then the reaction mixture warmed up to 30 °C and stirred for 1 h and extracted with EtOAc. The organic phase was washed with saturated $NaHCO_3$, brine, and dried over anhydrous Na_2SO_4, concentrated *in vacuo*,

and purified by flash column chromatography on silica gel to give an unseperatable mixture of **6** and thioglycoside byproduct totally 1.4 g as a yellow soap, which were used to next step directly without further purification.

chloromethyl-heptaacetyl-*β*-D-maltoside (**7**)

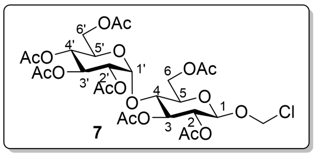

75.Mixture of compound **6** and thioglycoside byproduct (totally 1.4 g) was dissolved in dry DCM (18 mL) under argon atmosphere. Then SO_2Cl_2 (0.29 ml, 3.63 mmol) was added to the above solution at 0 °C. The reaction mixture stirred for 1 h and extracted with EtOAc. The organic phase was washed with saturated $NaHCO_3$, brine, and dried over anhydrous Na_2SO_4, concentrated *in vacuo*, and purified by flash column chromatography on silica gel (the silica gel was deactivated with 3% Et_3N in petroleum ether) to give **7** (580 mg, 28% for 2 steps) as a white soap. R*f* = 0.50 (petroleum ether-EtOAc 2:1). $[\alpha]_D^{25}$ +43.8 (*c*, 0.37 in $CHCl_3$). ^1H NMR (400 MHz, $CDCl_3$) δ 5.53 (d, *J* = 6.4 Hz, 1H, -OCH_2-), 5.44 (d, *J* = 6.4 Hz, 1H, -OCH_2-), 5.39 (d, *J* = 4.0 Hz, 1H, **H-1'**), 5.34 (t, *J* = 10.0 Hz, 1H, **H-3'**), 5.31 – 5.23 (m, 1H, **H-3**), 5.04 (t, *J* = 10.0 Hz, 1H, **H-4'**), 4.93 – 4.78 (m, 2H, **H-1, H-2**), 4.83 (dd, *J* = 9.6, 4.0 Hz, 1H, **H-2'**), 4.51 (dd, *J* = 12.0, 2.4 Hz, 1H, **H-6a**), 4.26 – 4.18 (m, 2H, **H-6a'**, **H-6b**), 4.06 – 3.98 (m, 2H, **H-6b'**, **H-4**), 3.93 (m, 1H, **H-5'**), 3.74 (m, 1H, **H-5**), 2.13 (s, 3H, -OAc), 2.08 (s, 3H, -OAc), 2.03 (s, 3H, -OAc), 2.02 (s, 3H, -OAc), 2.01 (s, 3H, -OAc), 1.99 (s, 3H, -OAc), 1.98 (s, 3H, -OAc). ^{13}C NMR (150 MHz, $CDCl_3$) δ 170.8, 170.8, 170.6, 170.3, 170.2, 169.9, 169.6, 96.4, 95.8, 75.2, 72.9, 72.5, 71.4, 70.2, 69.5, 68.8, 68.2, 62.6, 61.7, 21.1, 21.0, 20.9, 20.8, 20.8. HRMS (ESI-TOF) m/z: $[M+Na]^+$ calc. for $C_{27}H_{37}ClO_{18}Na$ 707.1561; found: 707.1554.

N-(4-(1,1,1,3,3,3-hexafluoro-2-(heptaacetyl-*β*-D-maltosyl)-2-yl)phenyl)-4-(trifluoromethyl)benzamide (**8**)

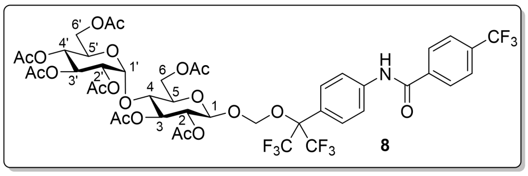

78.**SR1078** (292 mg, 0.66 mmol), ^{t}BuOK (86 mg, 0.77 mmol), TBAI (8 mg, 0.022 mmol) was dissolved in dry DMF (1.1 mL) in the present of 4Å MS (100 mg/mL) under argon atmosphere. After stirring 10 min at 0 °C, **7** (150 mg, 0.22 mmol) was dissolved in DMF (1.1 mL) and then added to above solution. Then the reaction mixture warmed up to 30 °C and stirred for 1 h and extracted with EtOAc. The organic phase was

washed with saturated brine, dried over anhydrous Na_2SO_4, concentrated *in vacuo*, and purified by flash column chromatography on silica gel to give **8** (146 mg, 62%) as a white soap. R*f* = 0.67 (petroleum ether-EtOAc 1:1). $[\alpha]_D^{25}$ +2.0 (*c*, 1.0 in $CHCl_3$). ^{1}H NMR (600 MHz, $CDCl_3$) δ 7.98 (d, *J* = 8.4 Hz, 2H), 7.95 (s, 1H, -NH-), 7.81 – 7.69 (m, 4H), 7.58 (d, *J* = 8.4 Hz, 2H), 5.40 (d, *J* = 4.2 Hz, 1H, **H-1'**), 5.35 (t, *J* = 10.2 Hz, 1H, **H-3'**), 5.30 (t, *J* = 9.0 Hz, 1H, **H-3**), 5.17 (d, *J* = 6.6 Hz, 1H, -OCH_2-), 5.04 (t, *J* = 9.6 Hz, 1H, **H-4'**), 4.95 (d, *J* = 8.4 Hz, 1H, **H-1**), 4.90 – 4.86 (dd, *J* = 9.0, 8.4 Hz, 1H, **H-2**), 4.85 – 4.82 (m, 2H, -OCH_2-, **H-2'**), 4.52 (dd, *J* = 12.0, 2.4 Hz, 1H, **H-6a**), 4.22 (m, 2H, **H-6b**, **H-6a'**), 4.05 – 4.00 (m, 2H, **H-4**, **H-6b'**), 3.96 – 3.92 (m, 1H, **H-5'**), 3.78 – 3.71 (m, 1H, **H-5**), 2.11 (s, 3H, -OAc), 2.08 (s, 3H, -OAc), 2.04 (s, 3H, -OAc), 2.04 (s, 3H, -OAc), 2.01 (s, 3H, -OAc), 2.00 (s, 3H, -OAc), 1.99 (s, 3H, -OAc). ^{13}C NMR (150 MHz, $CDCl_3$) δ = 170.6, 170.6, 170.5, 170.2, 170.0, 169.8, 169.4, 164.6, 139.7, 137.7, 134.0 (q, *J* = 33 Hz), 129.3, 127.6, 126.0, 126.0, 124.4, 123.6, 123.2, 122.6, 121.3, 121.1, 120.1, 96.8, 95.6, 88.5, 82.7 (q, *J* = 30 Hz), 75.2, 72.5, 72.4, 71.7, 70.0, 69.3, 68.5, 68.0, 62.5, 61.5, 29.7, 20.9, 20.8, 20.7, 20.6, 20.6, 20.4. ^{19}F NMR (565 MHz, $CDCl_3$) δ -63.04 (s), -70.81 (q, *J* = 9.9 Hz), -72.36 (q, *J* = 10.0 Hz). HRMS (ESI-TOF) m/z: $[M+Na]^+$ calc. for $C_{44}H_{46}F_9NO_{20}Na$ 1102.2362; found: 1102.2354.

N-(4-(1,1,1,3,3,3-hexafluoro-2-(*β*-D-maltosyl)-2-yl)phenyl)-4-(trifluoromethyl)benzamide (**Malt-SR**)

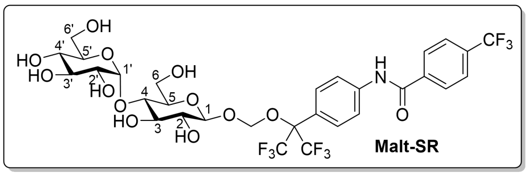

81.To a stirred solution of **8** (146 mg, 0.14 mmol) in MeOH (1.4 mL) was added K_2CO_3 (4 mg, 0.027 mmol) at room temperature. The mixture was stirred overnight and directly purified by column chromatography on Sephadex LH-20 to afford compound **Malt-SR** (100 mg, 94%) as a white a crystal. R*f* = 0.25 (DCM-MeOH 8:1). m.p. 124.4-129.5 °C. $[\alpha]_D^{25}$ +80.7 (*c*, 0.46 in CH_3OH). ^{1}H NMR (600 MHz, CD_3OD) δ 8.12 (d, *J* = 7.8 Hz, 2H), 7.92 (d, *J* = 9.0 Hz, 2H), 7.84 (d, *J* = 8.4 Hz, 2H), 7.74 (d, *J* = 8.4 Hz, 2H), 5.29 (d, *J* = 5.4 Hz, 1H, -OCH_2-), 5.18 (d, *J* = 4.2 Hz, 1H, **H-1'**), 4.95 (d, *J* = 5.4 Hz, 1H, -OCH_2-), 4.70 (d, *J* = 7.8 Hz, 1H, **H-1**), 3.91 – 3.87 (m, 1H, **H-6a**), 3.84 – 3.82 (m, 2H, **H-6b**, **H-6a'**), 3.71 – 3.64 (m, 3H, **H-3**, **H-5'**, **H-6b'**), 3.62 (t, *J* = 9.6 Hz, 1H, **H-3'**), 3.58 (t, *J* = 9.0 Hz, 1H, **H-4**), 3.48 – 3.43 (m, 2H, **H-5**, **H-2'**), 3.32 – 3.2 (m, 1H, **H-2**), 3.27 (t, *J* = 9.6 Hz, 1H, **H-4'**). ^{13}C NMR (150 MHz, CD_3OD) δ 167.5, 142.1, 139.8, 134.4 (q, *J* = 32.4 Hz), 130.3, 129.5, 126.6 (q, *J* = 3.3 Hz), 125.3

(q, J = 270.2 Hz), 124.5, 121.9, 102.9, 101.3, 90.3, 83.5 (q, J = 29.1 Hz), 80.9, 77.6, 76.9, 75.1, 74.8, 74.5, 74.2, 71.5, 62.8, 62.0. ^{19}F NMR (565 MHz, CD_3OD) δ -249.62 (s), -257.86 (q, J = 9.5 Hz), -257.98 (q, J = 9.5 Hz). HRMS (ESI-TOF) m/z: $[M+Na]^+$ calc. for $C_{30}H_{32}F_9NO_{13}Na$ 808.1622; found: 808.1619.

N-(4-(1,1,1,3,3,3-hexafluoro-2-hydroxypropan-2-yl)phenyl)-4-methylbenzamide (**9**)

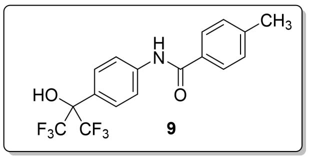

84.2-(4-aminophenyl)-1,1,1,3,3,3-hexafluoropropan-2-ol (100 mg, 0.39 mmol) was dissolved in DCM (1.5 mL) under argon atmosphere. 4-methylbenzoyl chloride (51 uL, 0.39 mmol) and DIPEA (80 uL, 0.46 mmol) was added to the above solution at 0 ºC. After stirring 10 min at 0 ºC, the reaction mixture warmed up to 30 °C and stirred for 2 h and extracted with EtOAc. The organic phase was washed with saturated brine, dried over anhydrous Na_2SO_4, concentrated *in vacuo*. The crude product was recrystallized with EtOAc/petroleum ether and filtration, the compound **9** (111 mg, 77%) was obtained as a white solid. R*f* = 0.37 (petroleum ether-EtOAc 2:1). m.p. 156.7-163.2 °C. ^{1}H NMR (400 MHz, DMSO-*d*6) δ 10.37 (s, 1H), 8.64 (s, 1H), 7.90 (m, 4H), 7.65 (d, J = 8.8 Hz, 2H), 7.35 (d, J = 8.0 Hz, 2H), 2.39 (s, 3H). ^{13}C NMR (150 MHz, DMSO-*d*6) δ 165.6, 141.9, 140.8, 131.8, 129.0, 127.80 127.2, 125.3, 123.0 (q, J = 287.4 Hz), 120.0, 76.7 (q, J = 28.5 Hz), 21.1. ^{19}F NMR (565 MHz, DMSO-*d*6) δ -259.21 (s). HRMS (ESI-TOF) m/z: $[M+Na]^+$ calc. for $C_{17}H_{13}F_6NO_2Na$ 400.0743, found: 400.0733.

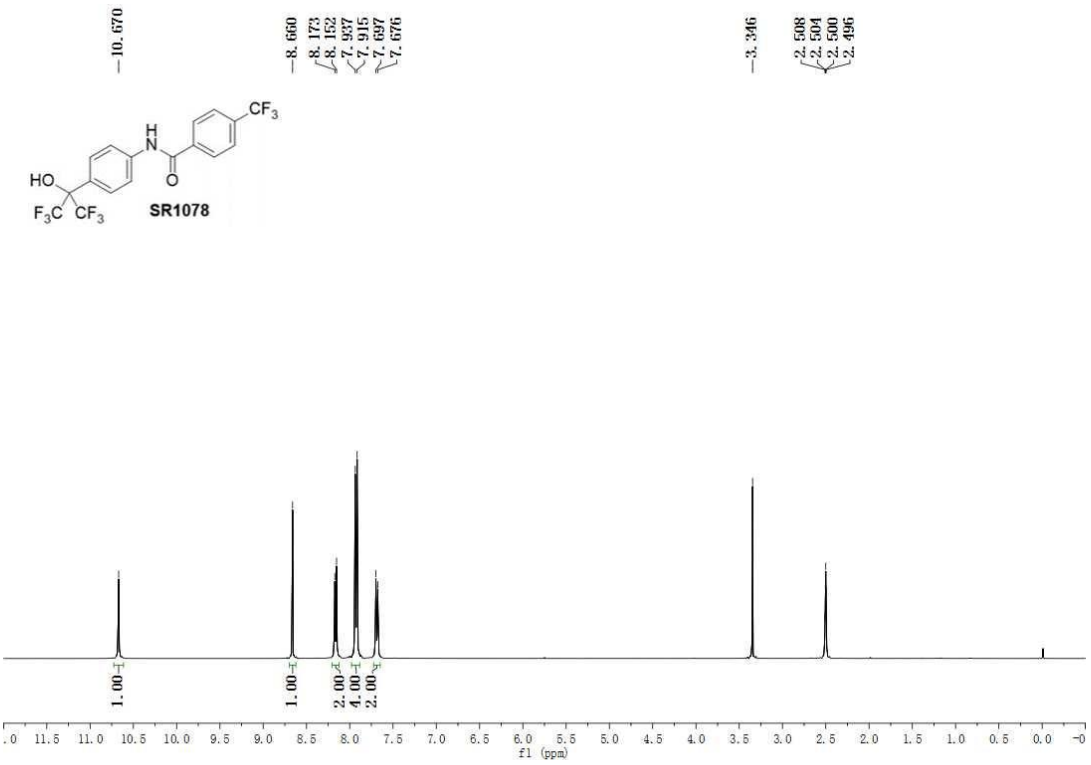

Figure S1. ^{1}H NMR (400 MHz, DMSO-*d6*) spectrum of **SR1078**

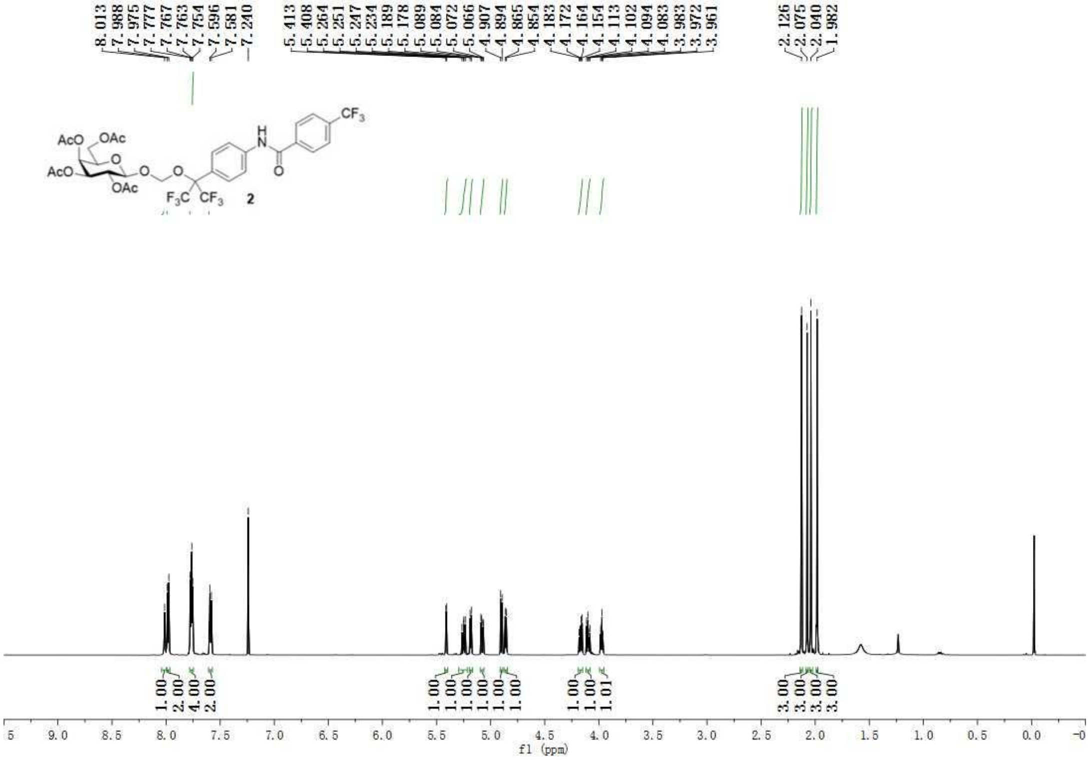

Figure S2. ^{1}H NMR (600 MHz, $CDCl_3$) spectrum of **2**

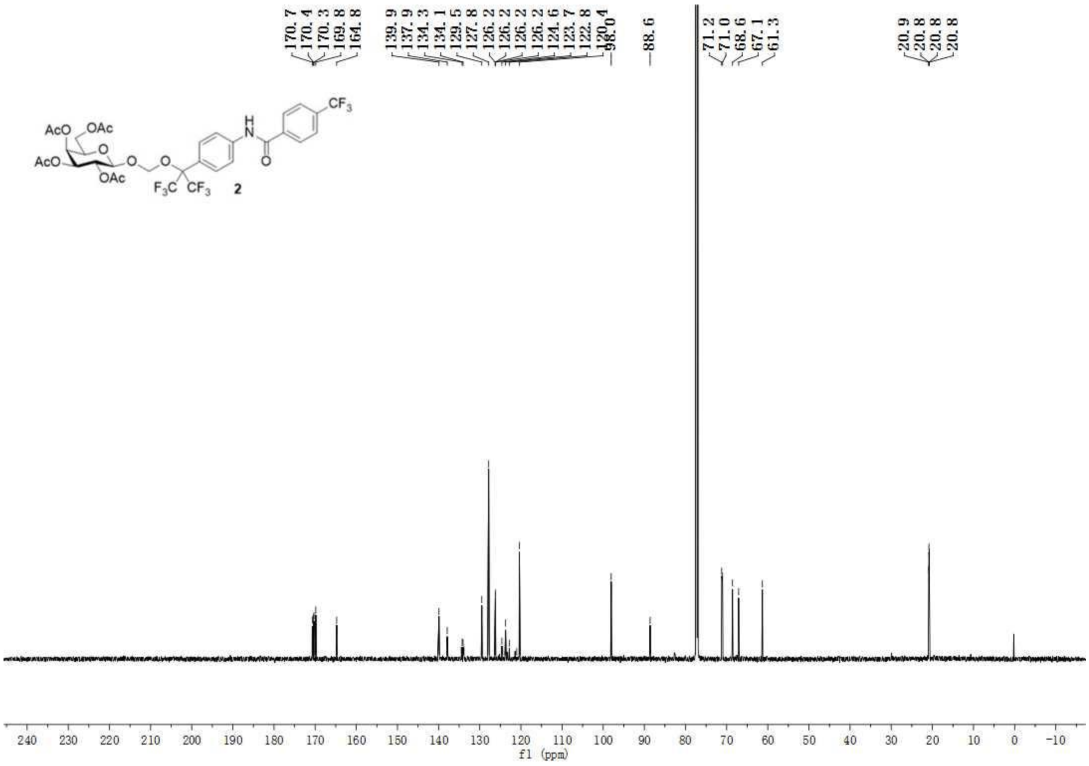

Figure S3. ^{13}C NMR (150 MHz, $CDCl_3$) spectrum of **2**

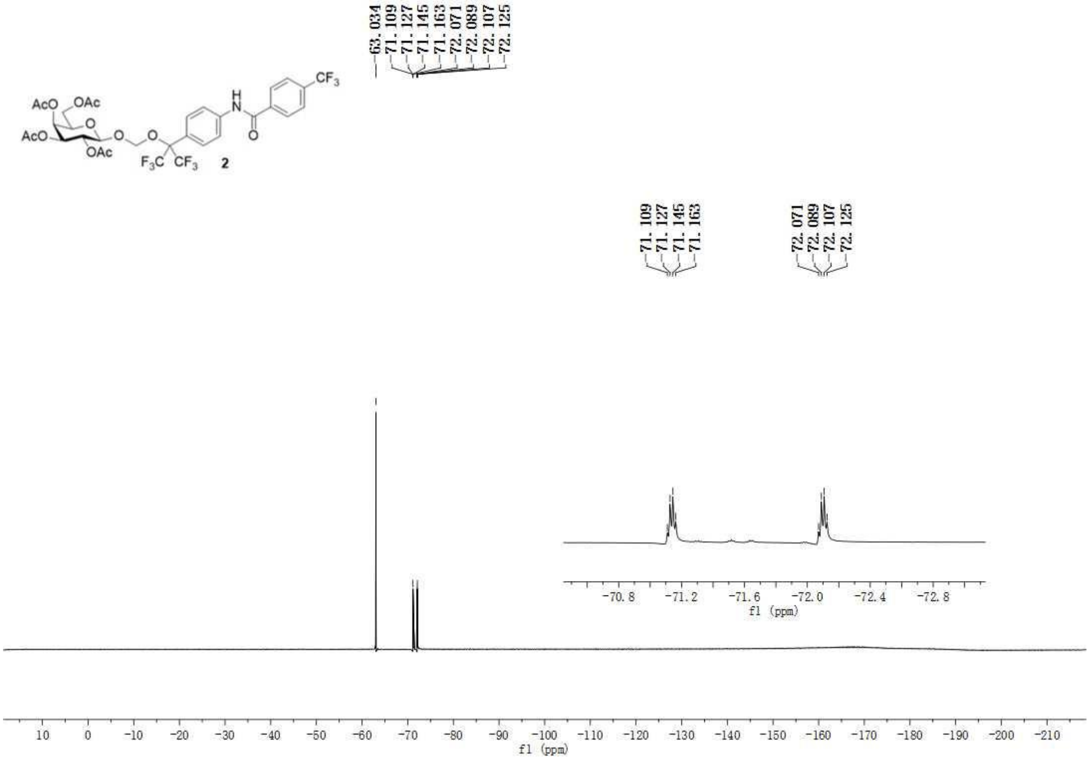

Figure S4. ^{19}F NMR (565MHz, $CDCl_3$) spectrum of **2**

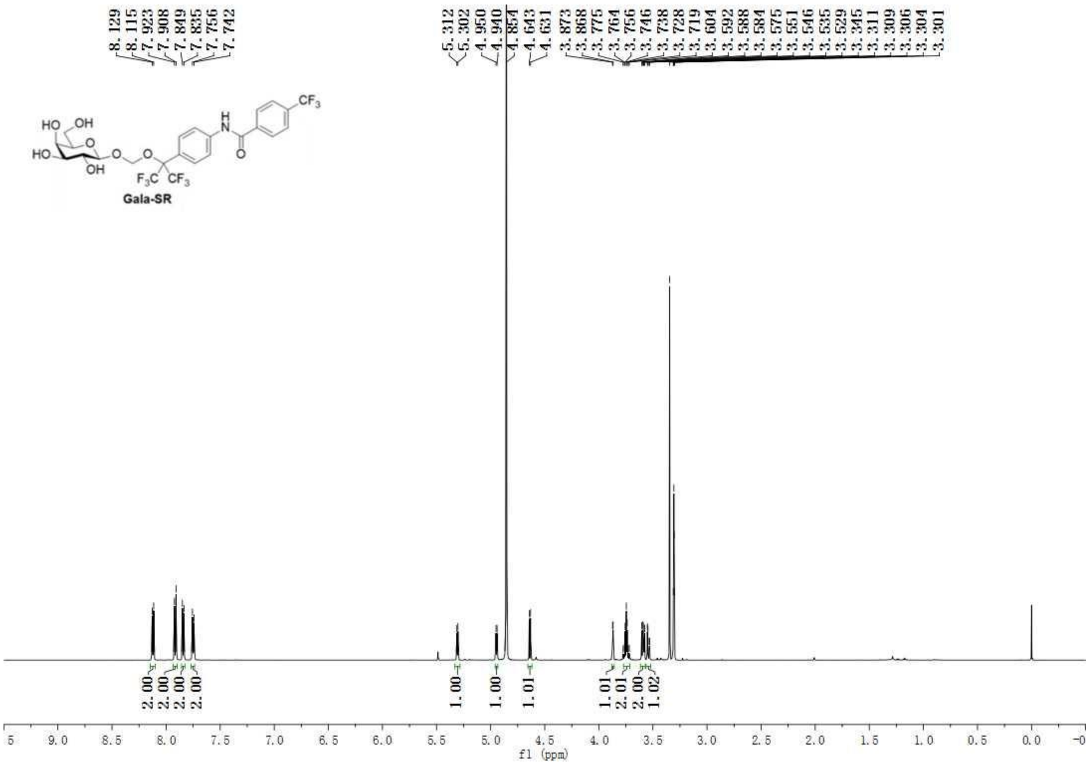

Figure S5. ^{1}H NMR (600 MHz, CD_3OD) spectrum of **Gala-SR**

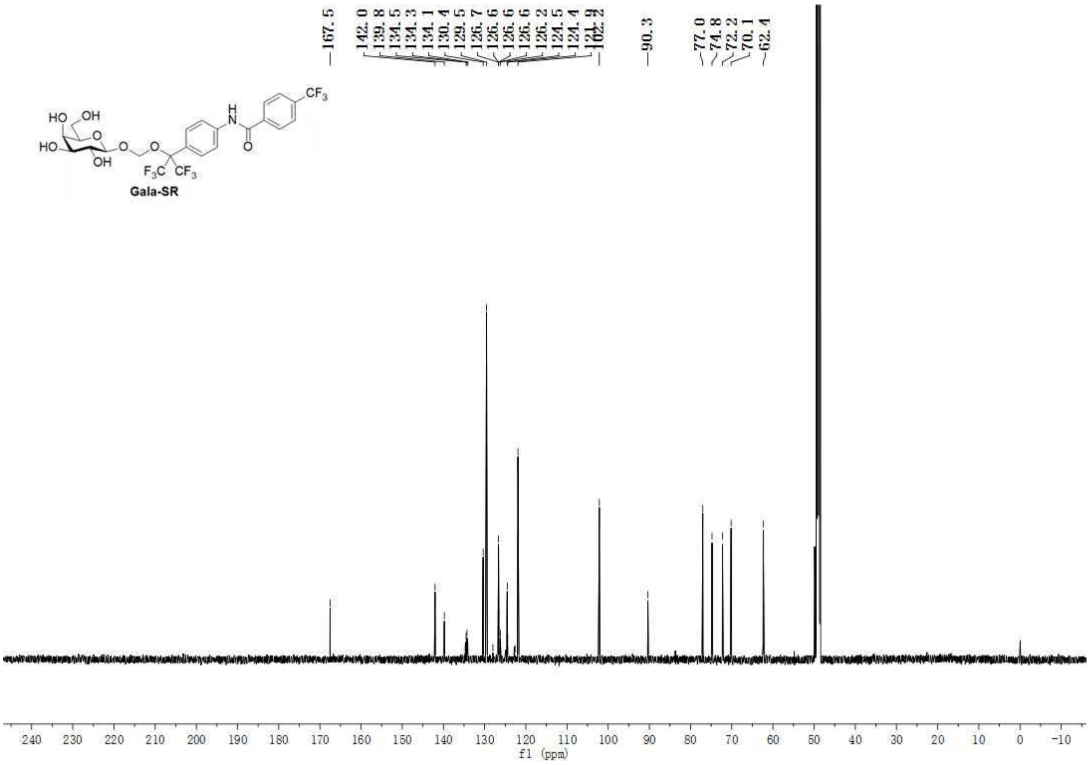

Figure S6. ^{13}C NMR (150 MHz, CD_3OD) spectrum of **Gala-SR**

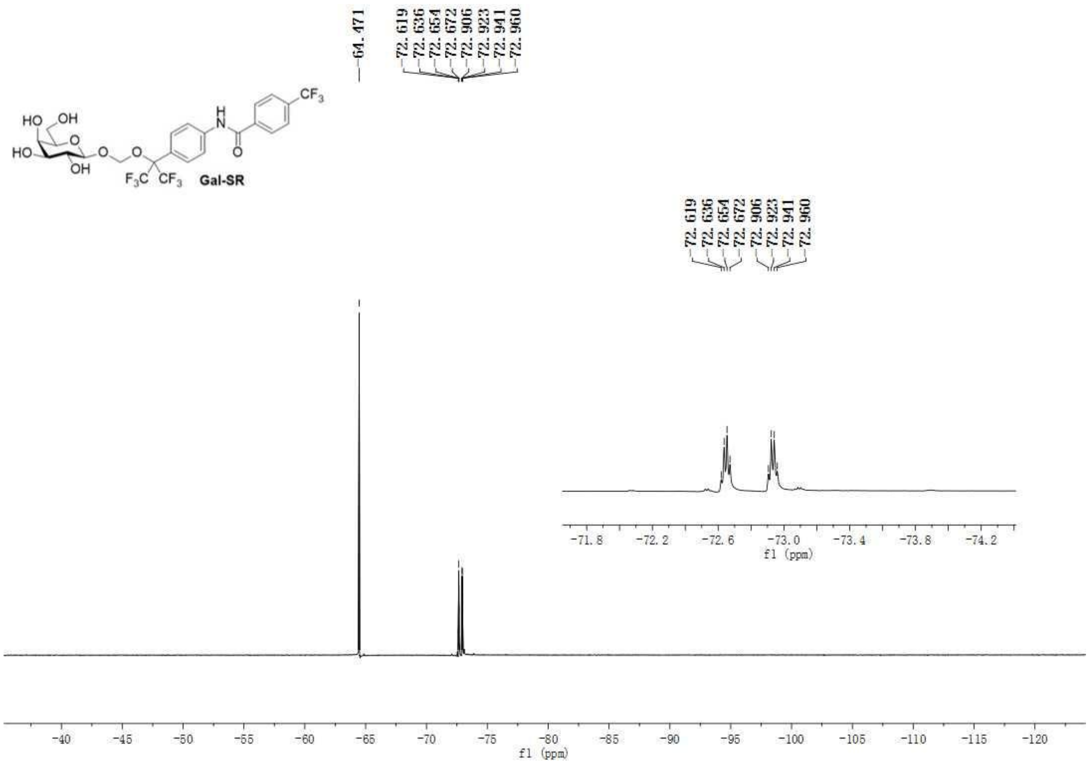

Figure S7. ^{19}F NMR (565MHz, CD_3OD) spectrum of **Gala-SR**

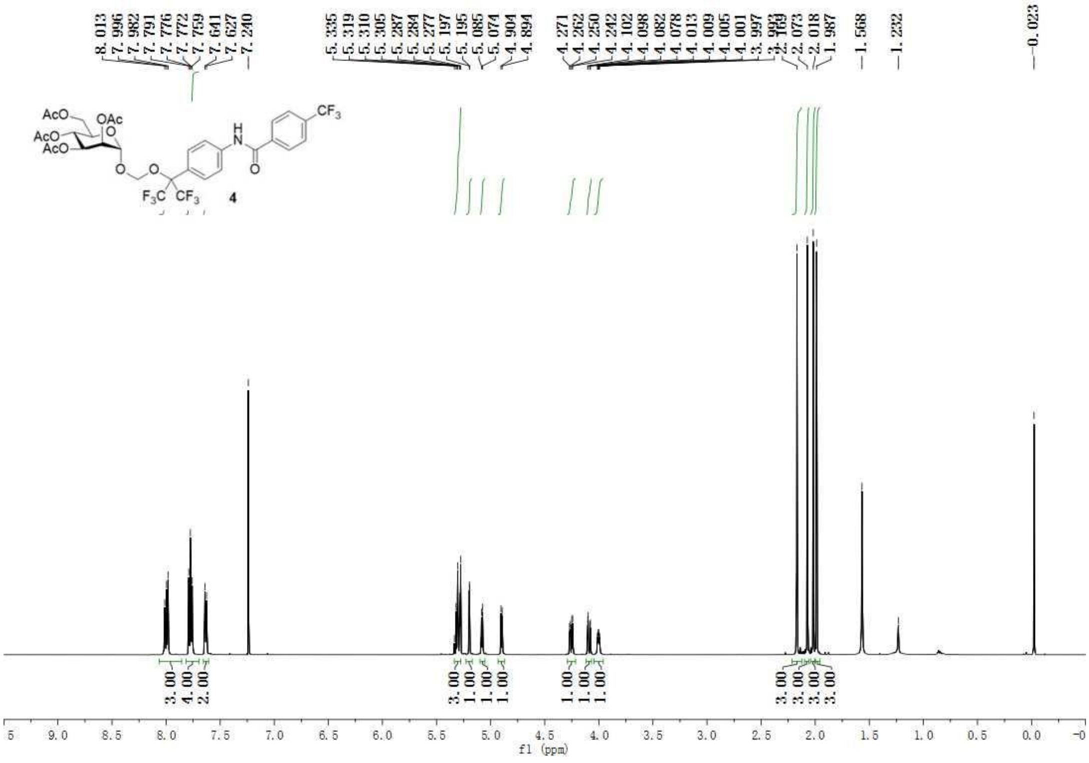

Figure S8. ^{1}H NMR (600 MHz, $CDCl_3$) spectrum of **4**

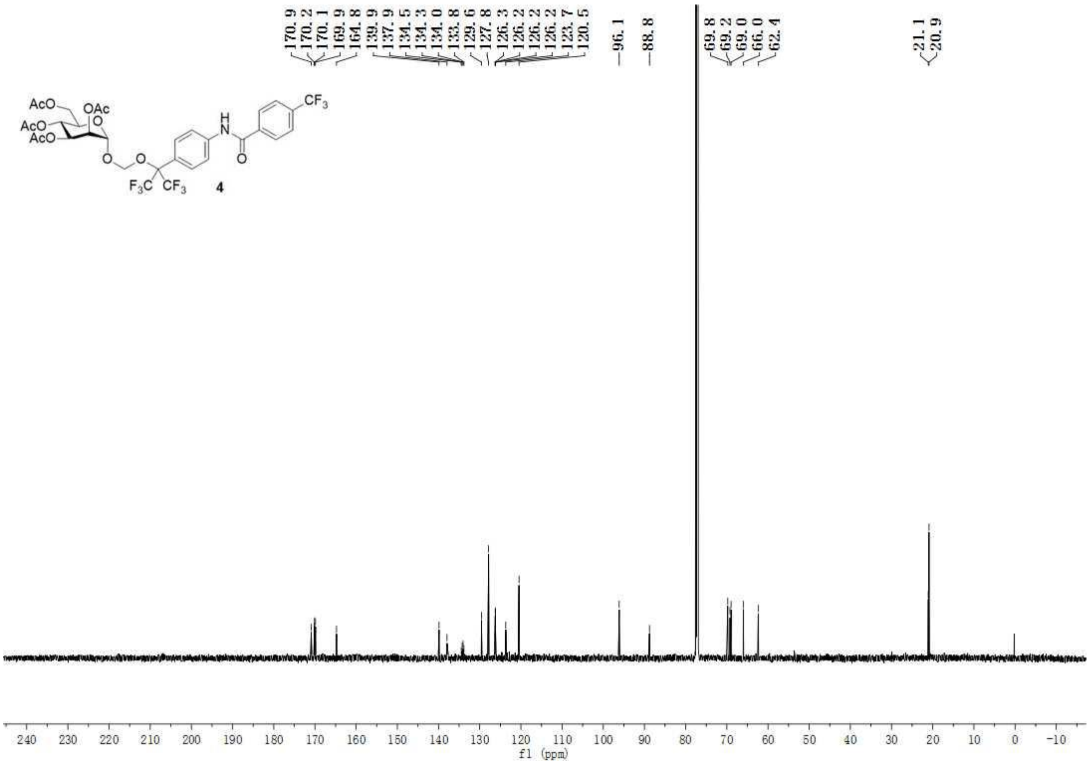

Figure S9. ^{13}C NMR (150 MHz, $CDCl_3$) spectrum of **4**

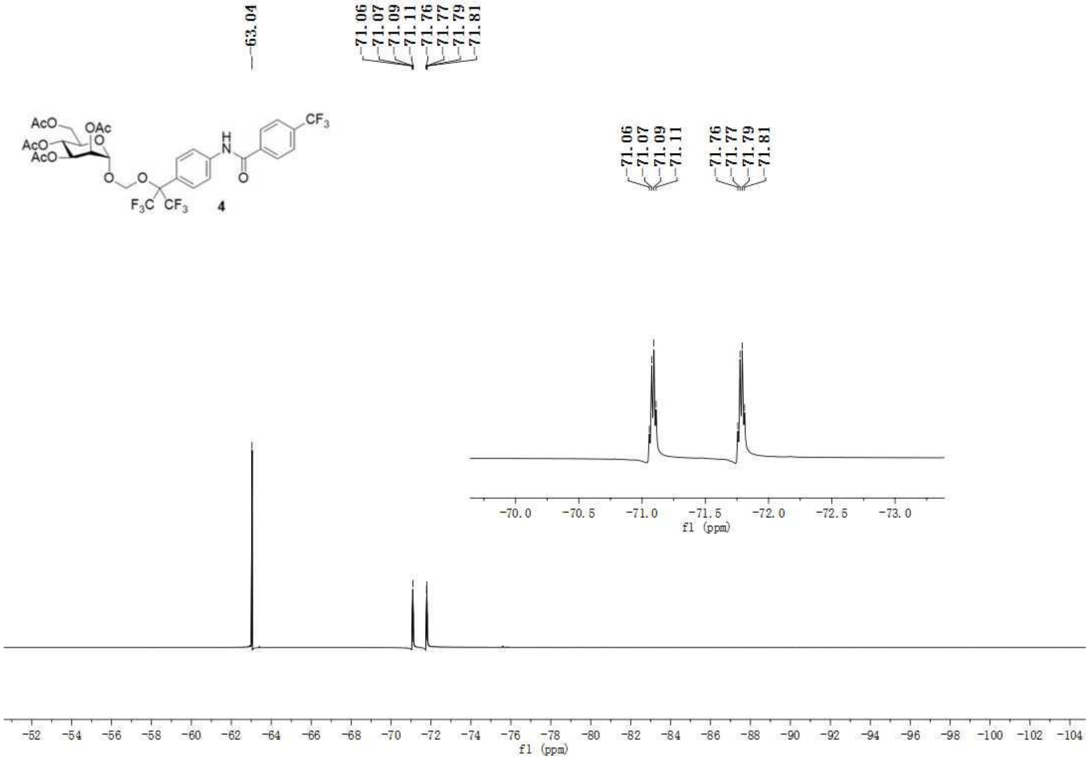

Figure S10. ^{19}F NMR (565 MHz, $CDCl_3$) spectrum of **4**

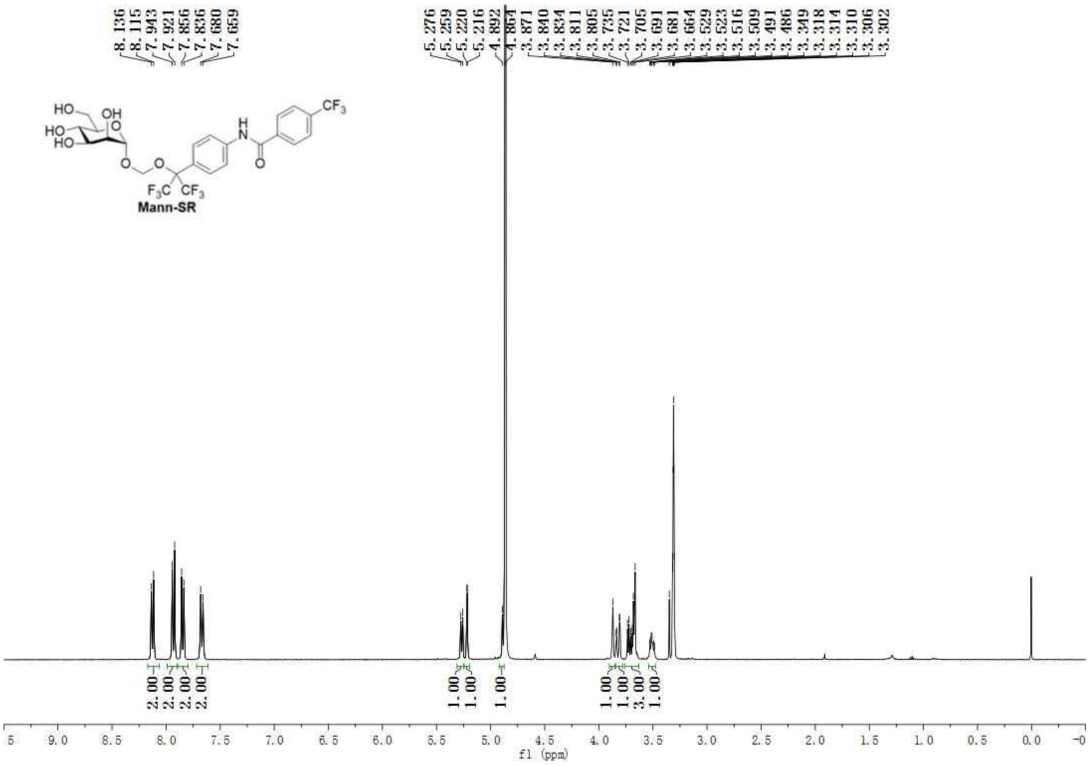

Figure S11. ^{1}H NMR (600 MHz, CD_3OD) spectrum of **Mann-SR**

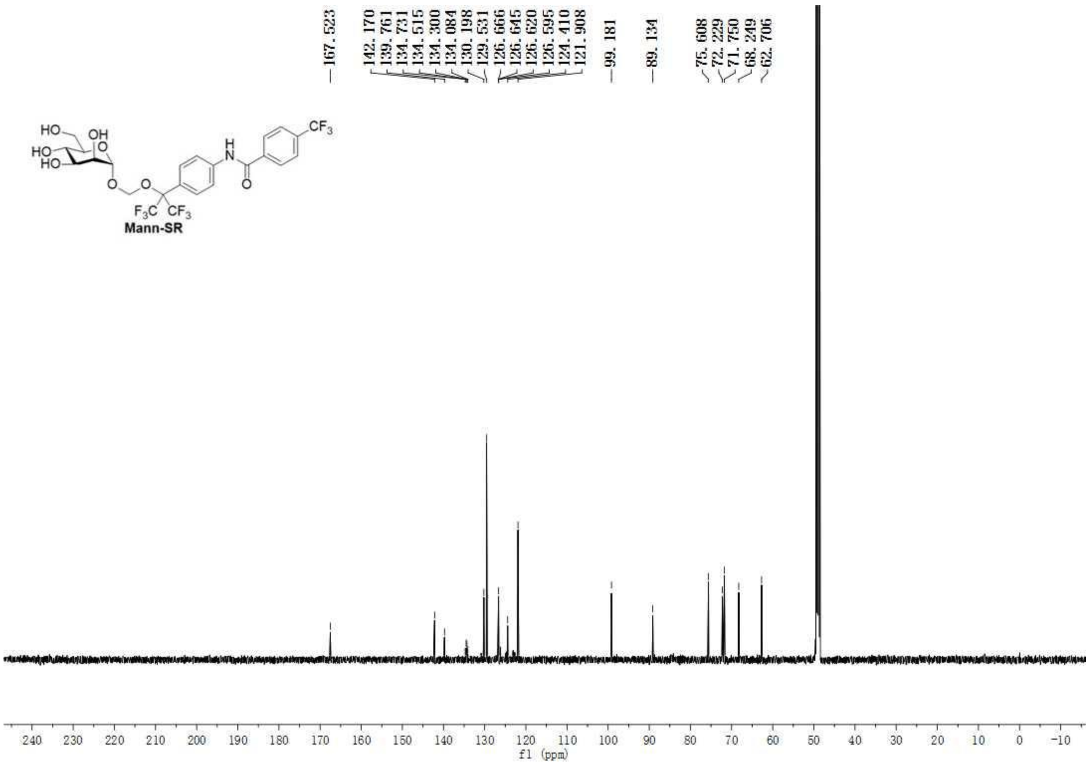

Figure S12. ^{13}C NMR (150 MHz, CD_3OD) spectrum of **Mann-SR**

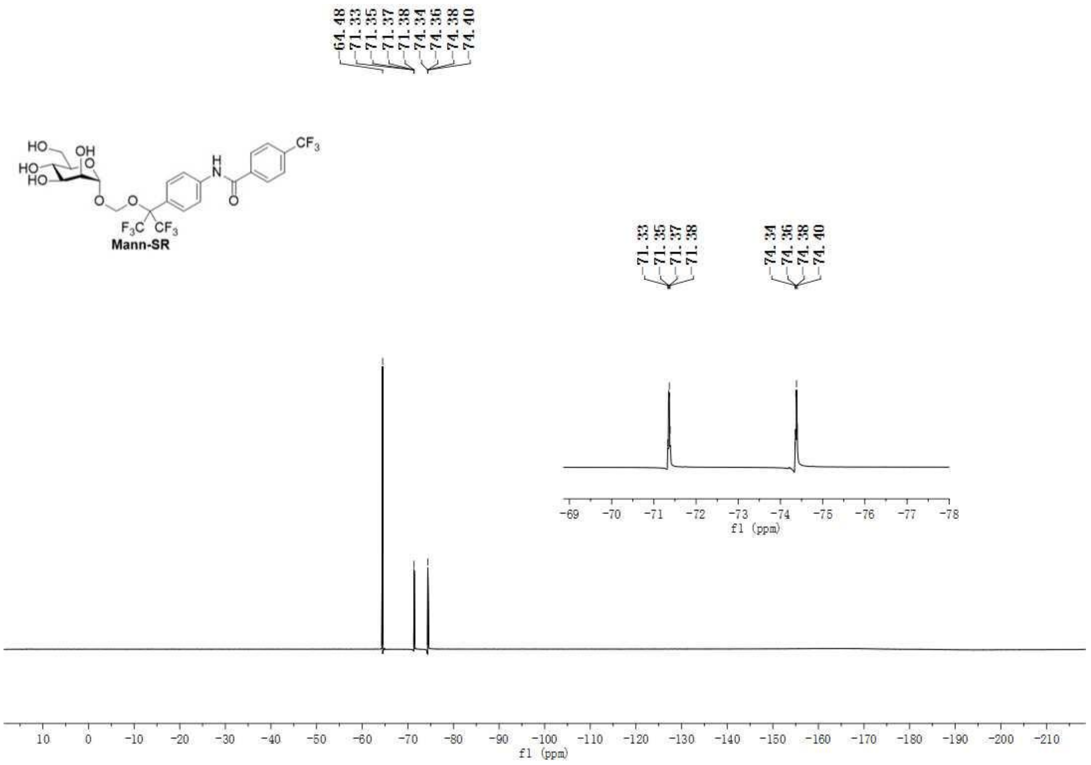

Figure S13. ^{19}F NMR (565 MHz, CD_3OD) spectrum of **Mann-SR**

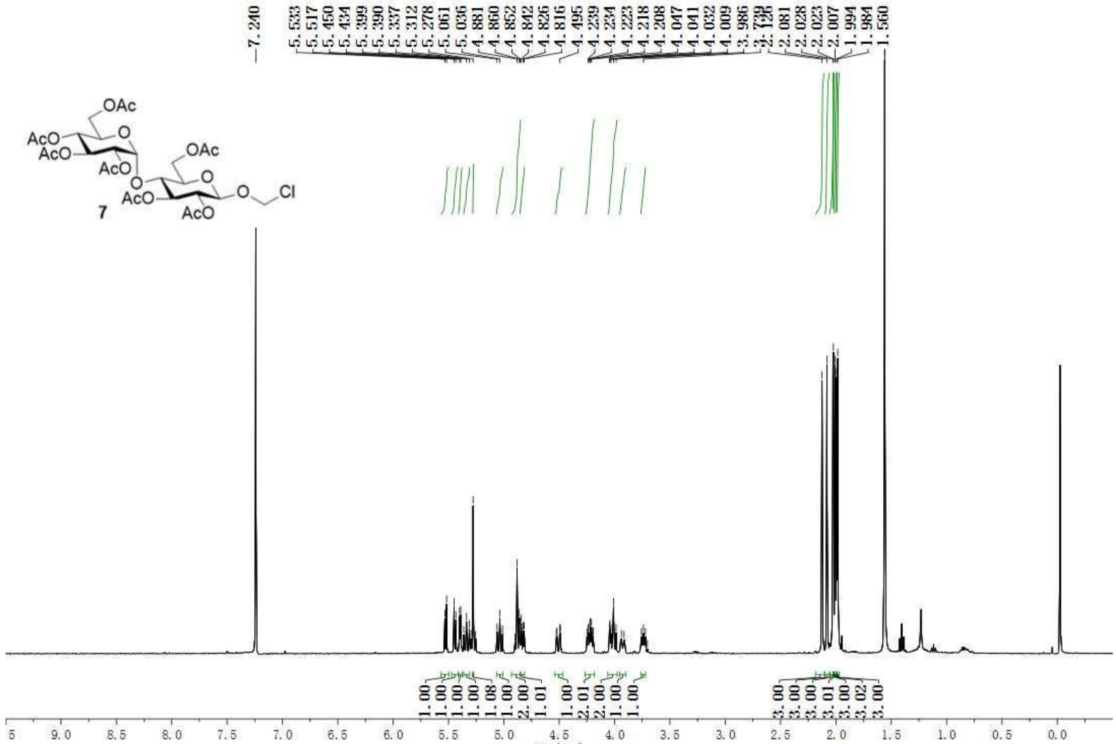

Figure S14. ^{1}H NMR (400 MHz, $CDCl_3$) spectrum of **7**

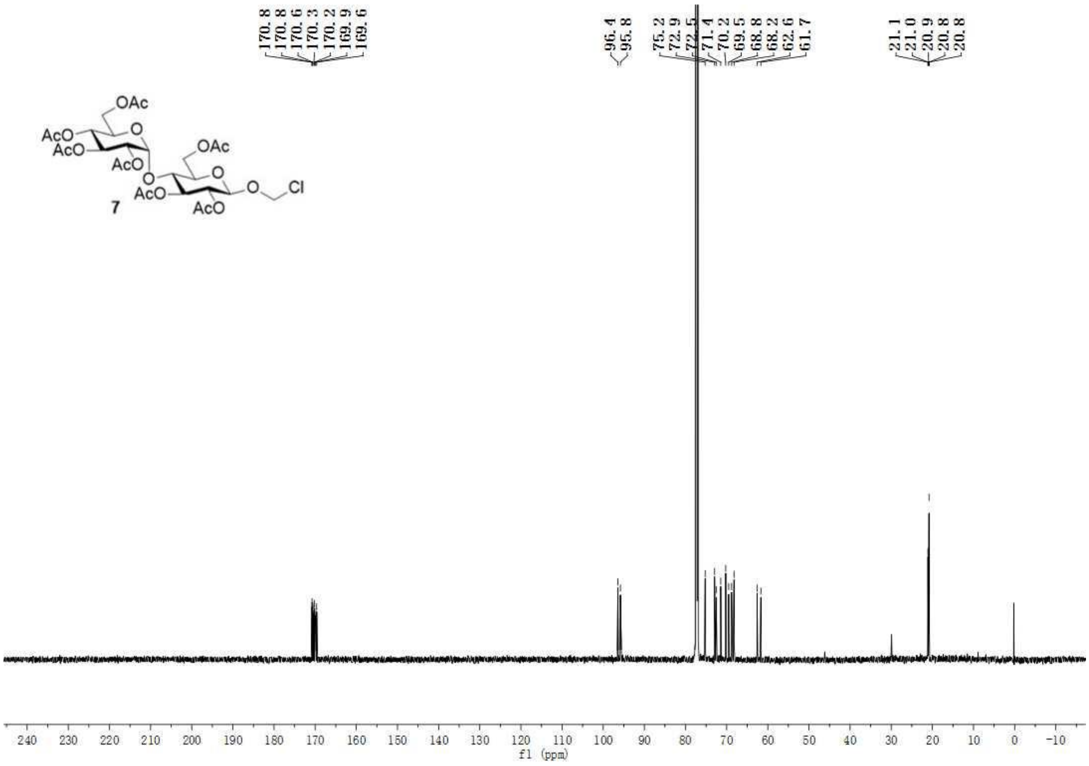

Figure S15. ^{13}C NMR (150 MHz, $CDCl_3$) spectrum of **7**

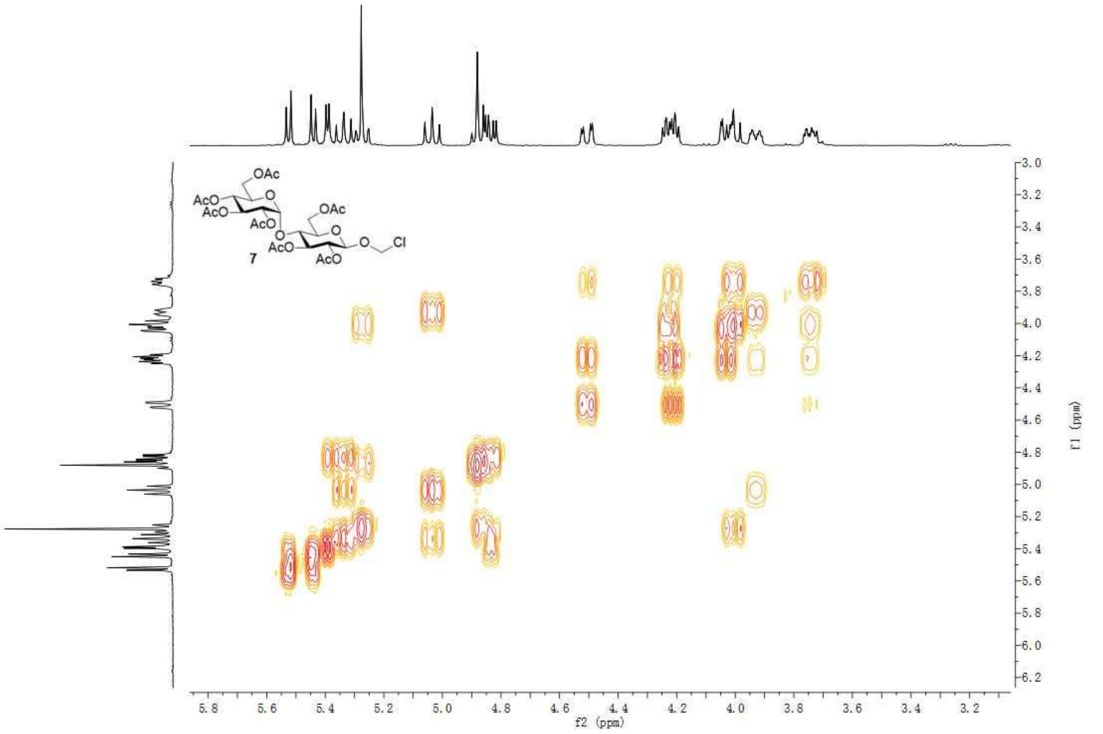

Figure S16. ^{1}H-^{1}HCOSY NMR (400 MHz, $CDCl_3$) spectrum of **7**

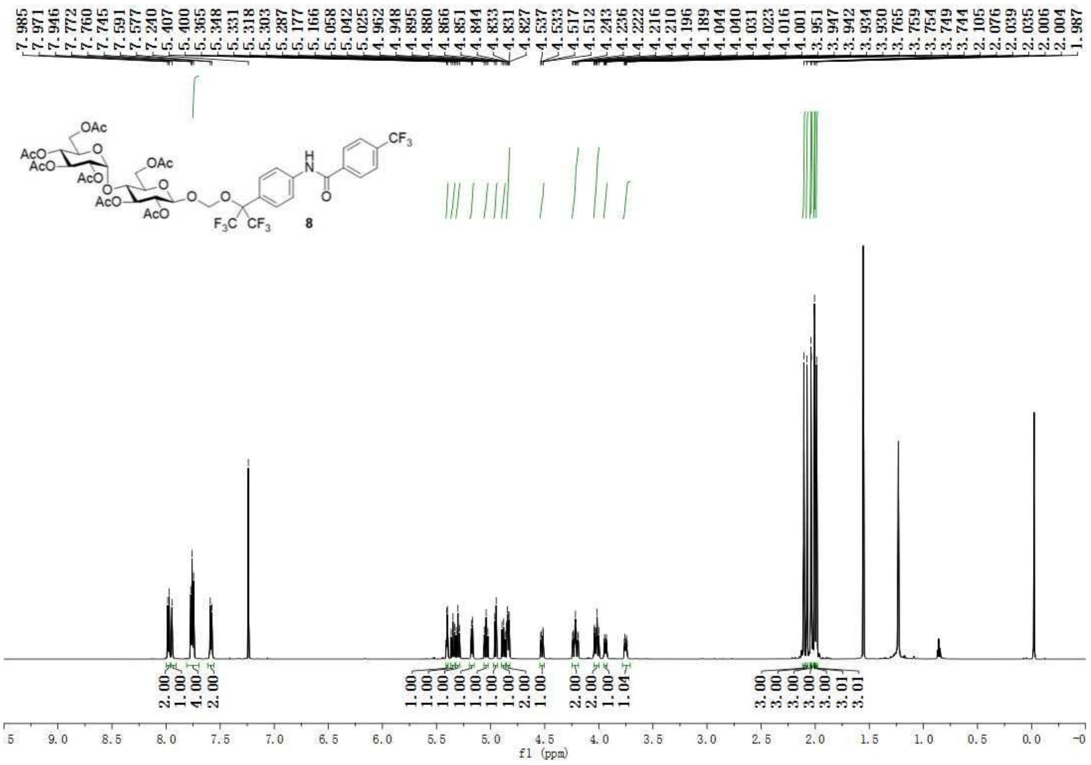

Figure S17. ^{1}H NMR (600 MHz, $CDCl_3$) spectrum of **8**

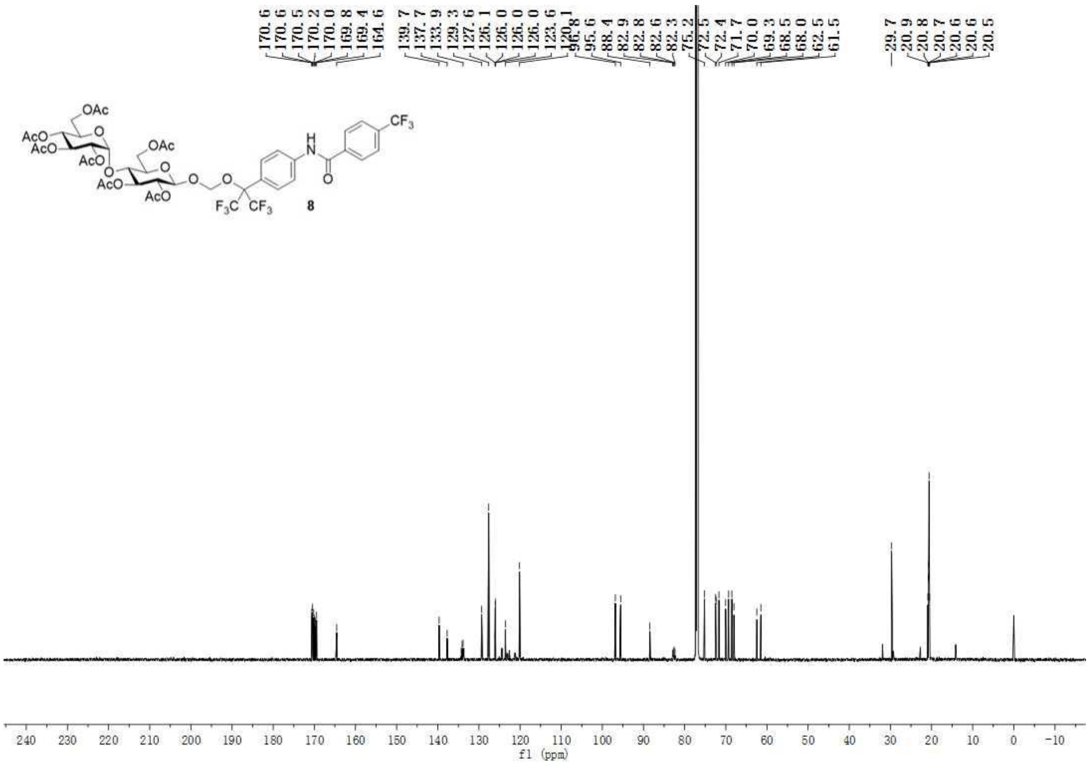

Figure S18. ^{13}C NMR (150 MHz, $CDCl_3$) spectrum of **8**

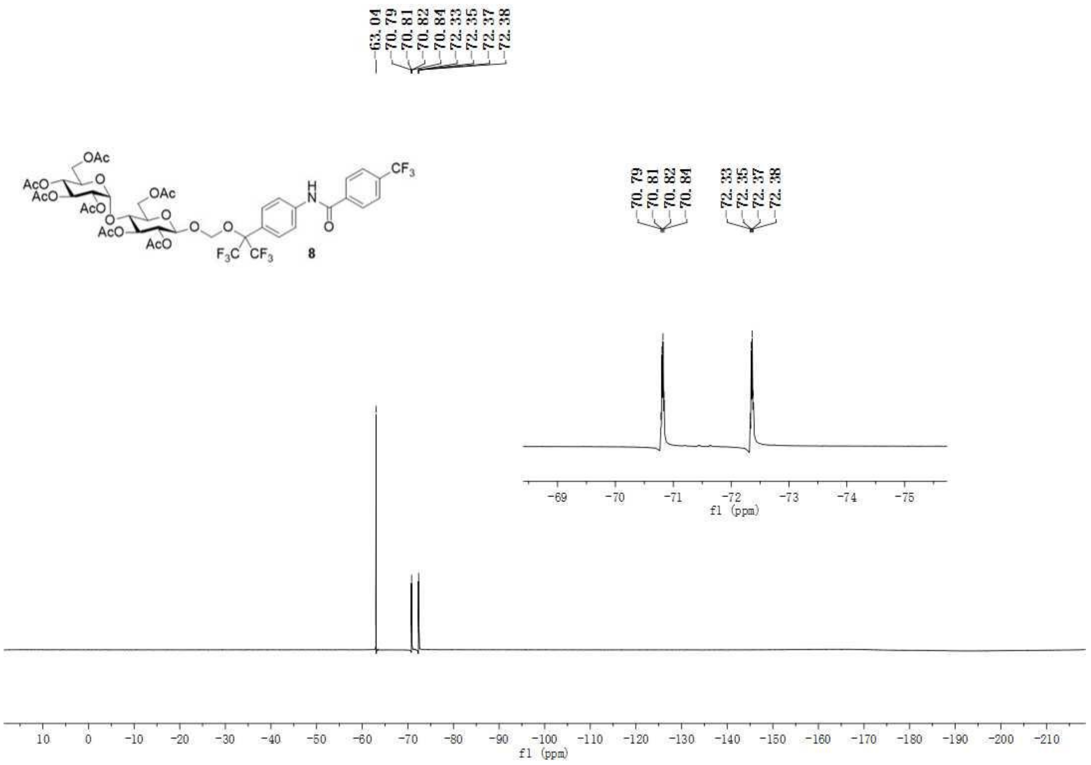

116.Figure S19. ^{19}F NMR (565 MHz, $CDCl_3$) spectrum of **8**

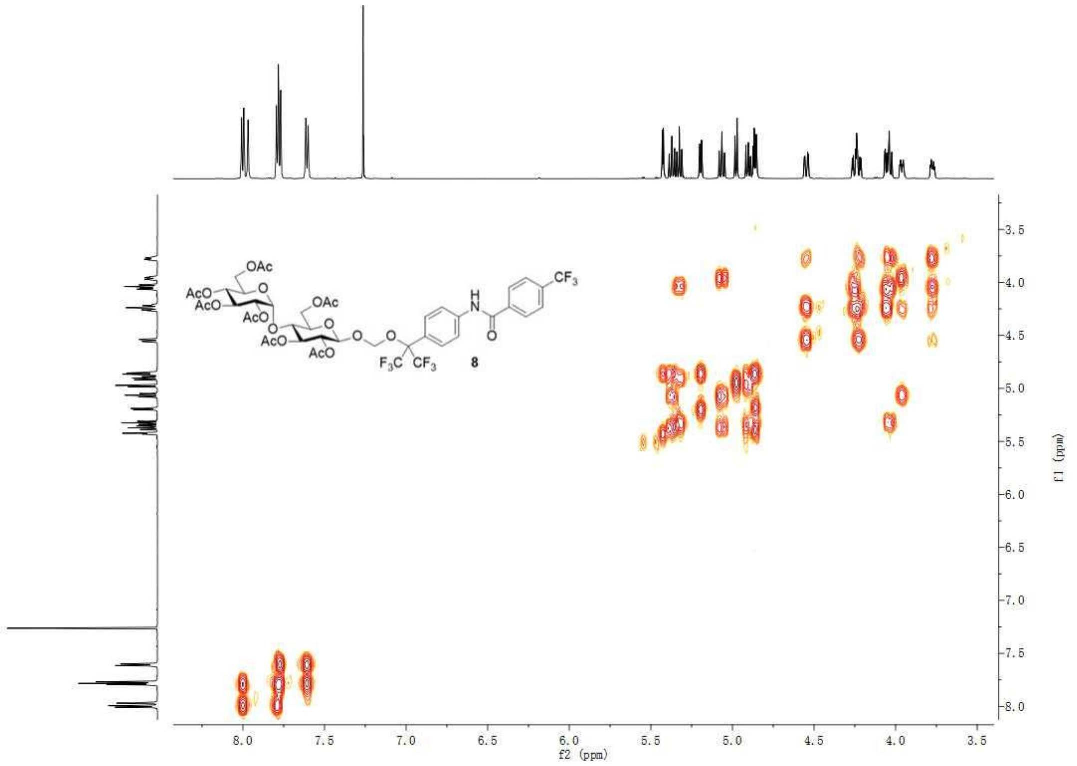

Figure S20. ^{1}H-^{1}HCOSY NMR (600 MHz, $CDCl_3$) spectrum of **8**

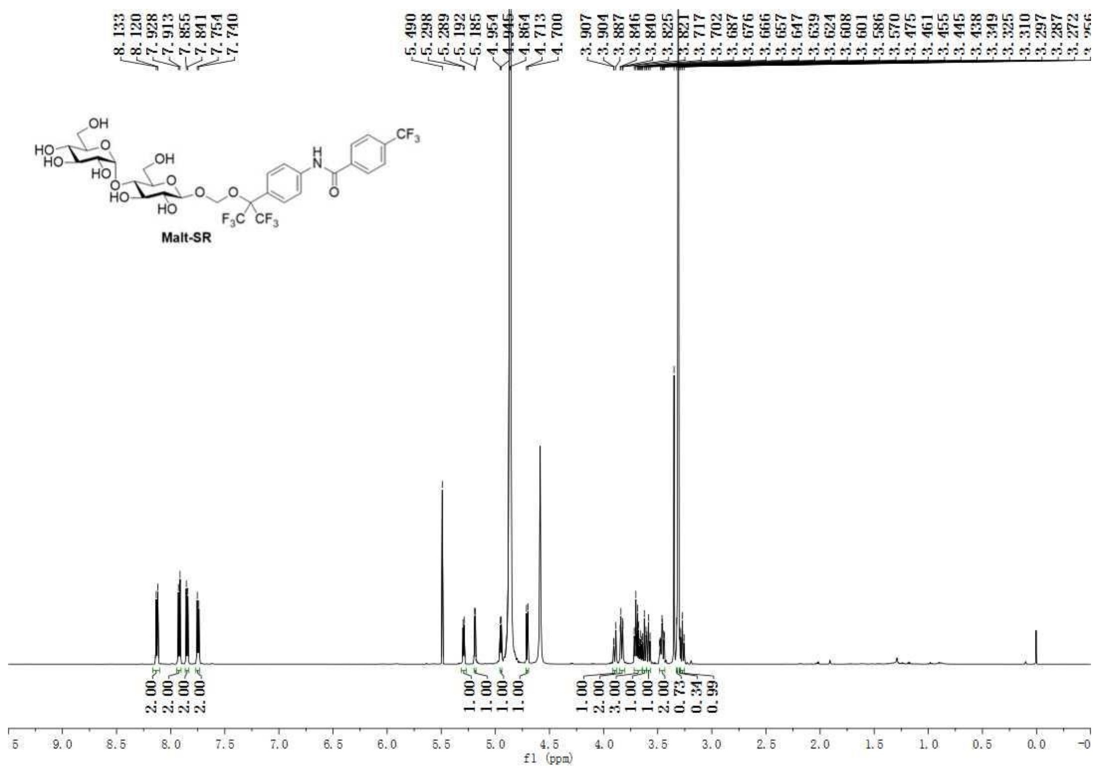

Figure S21. ^{1}H NMR (600 MHz, CD_3OD) spectrum of **Malt-SR**

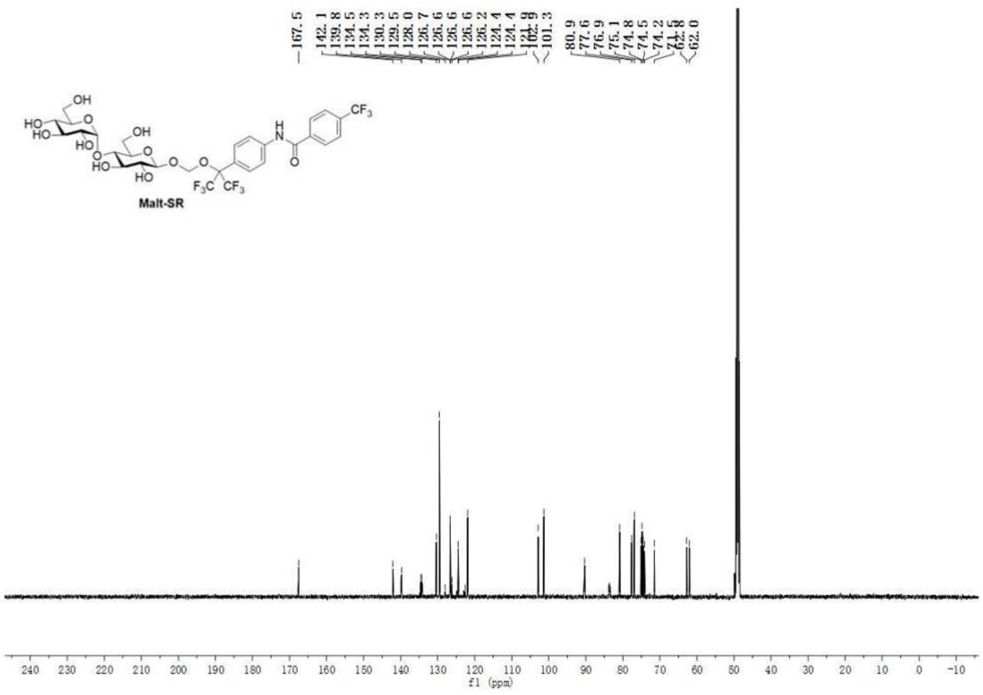

Figure S22. ^{13}C NMR (150 MHz, CD_3OD) spectrum of **Malt-SR**

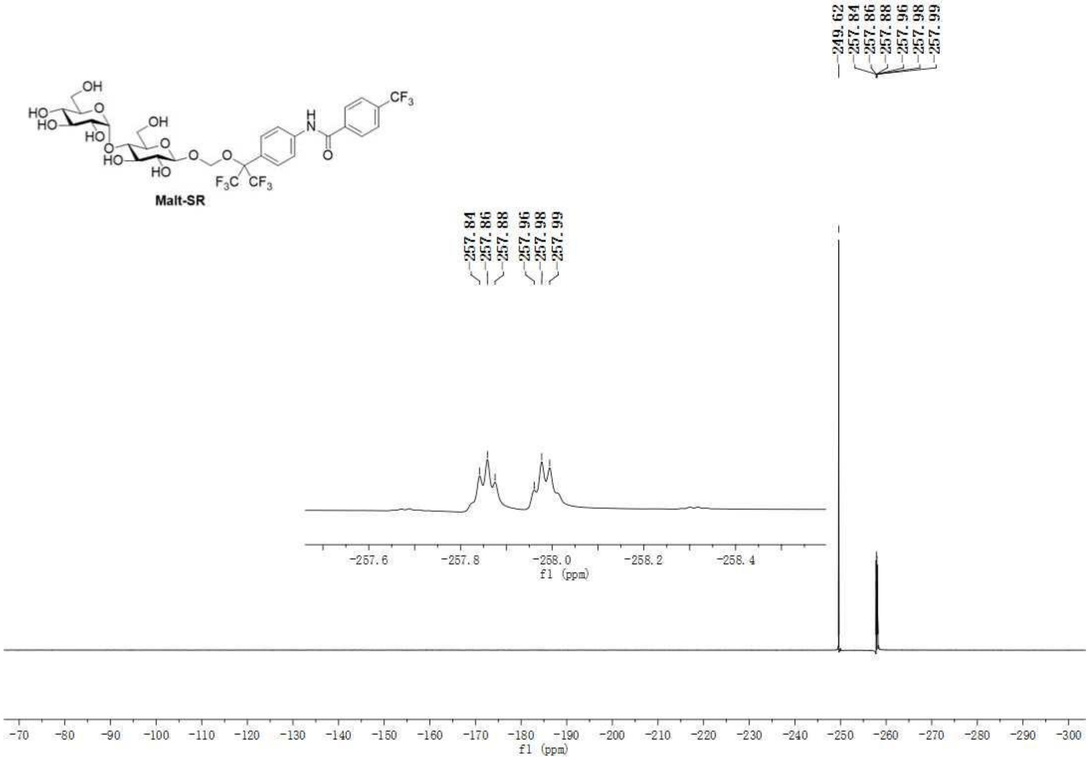

Figure S23. ^{19}F NMR (565 MHz, CD_3OD) spectrum of **Malt-SR**

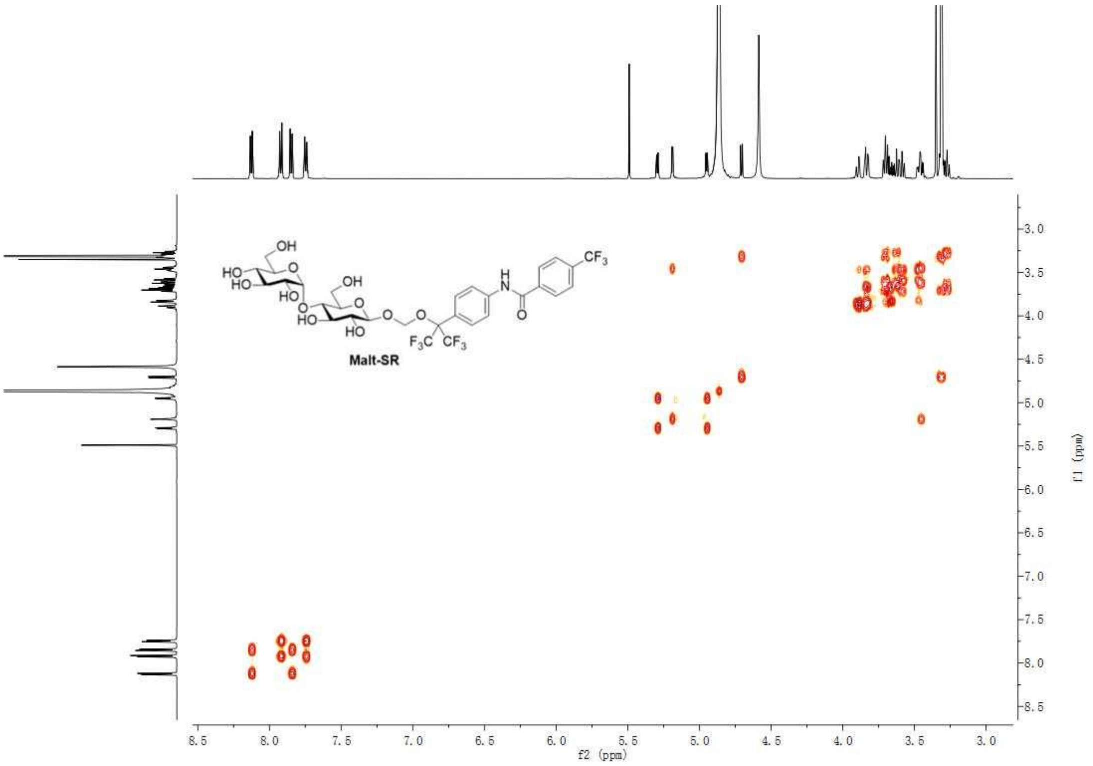

Figure S24. ^{1}H-^{1}HCOSY NMR (600 MHz, CD_3OD) spectrum of **Malt-SR**

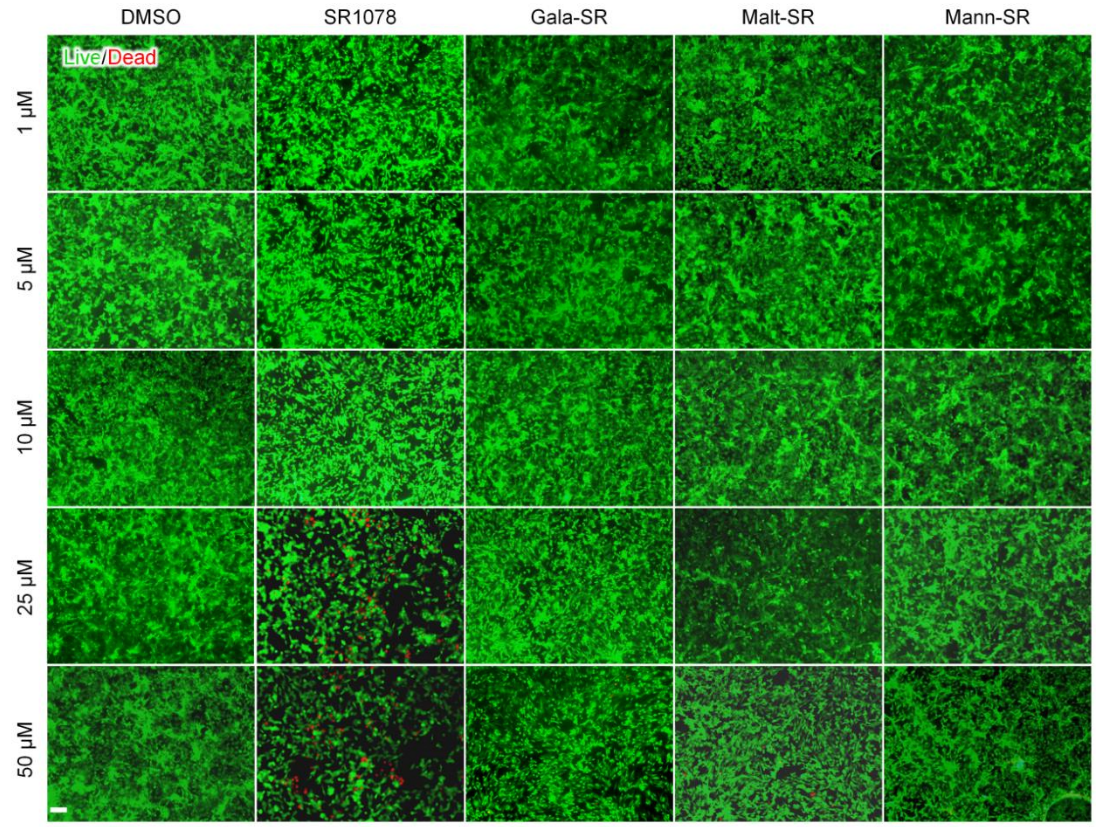

Figure S25. After U2OS cells were treated with various concentrations (0 ~ 50 μM) of SR1078, Gala-SR, Malt-SR, and Mann-SR for 24 hours, live and dead cells were labeled using the Calcein-AM/PI double-staining method. Scale bar: 50 μm.

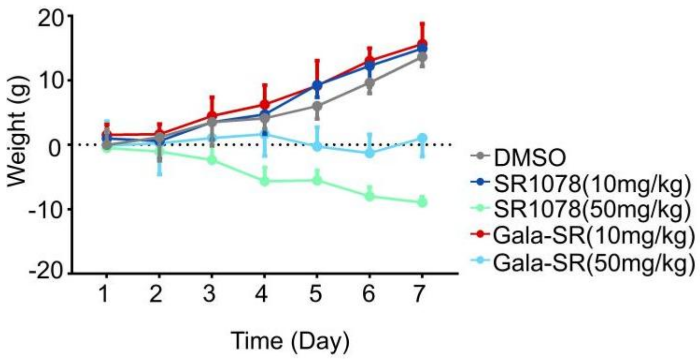

Figure S26.Galactose modification improves biocompatibility *in vivo*. Body weight changes (%) in C57BL/6J mice (n = 4) after 7 days of administration of Gala-SR, SR1078, or DMSO at doses of 10 mg/kg and 50 mg/kg via IP were measured.

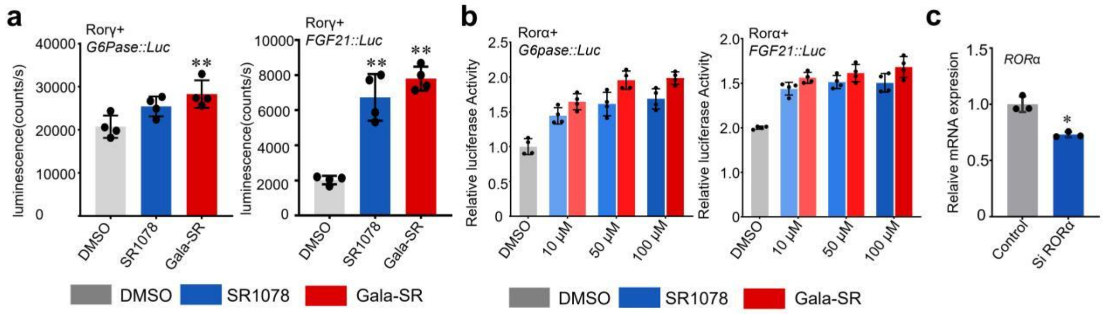

Figure S27. Galactose modification dose-dependently activates RORs-directed transcription. a. Cotransfection of HEK293T cells with RORγ and a reporter consisting of the *G6Pase* or *FGF21* promoters upstream of a luciferase reporter gene result in transcriptional stimulation upon addition of 5 μM SR1078 and Gala-SR treatments. (* represents the comparison with DMSO, **P<0.01, n=4). b. Cotransfection of HEK293T cells with RORα and a reporter construct (containing the G6Pase or FGF21 promoters upstream of a luciferase reporter gene) resulted in transcriptional stimulation upon treatment with SR1078 and Gala-SR (at concentrations of 0～100 μM; n=4). c. qPCR analyses was performed to determine mRNA expression of RORα after transfecting 293T cells with siRORα plasmid. (* represents the comparison with control *P<0.05, n=3).

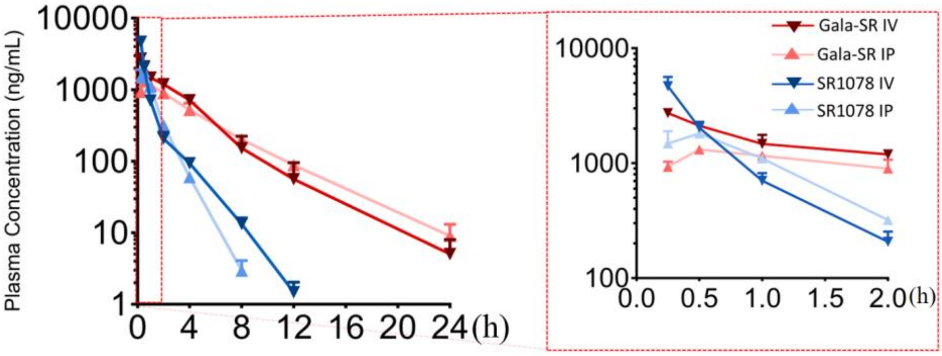

Figure S28. Gala-SR improved pharmacokinetic characteristics. Plasma concentration-time curves were obtained for Gala-SR and SR1078 after intraperitoneal (IP) and intravenous (IV) injection at a dose of 10 mg/kg in mice (n=3).

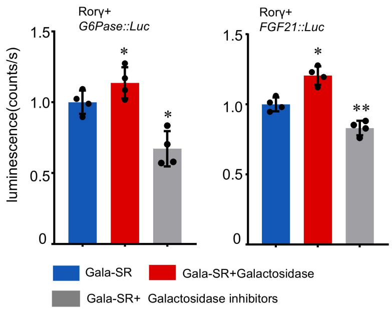

Figure S29. Exploring the impact of acetal linker design on RORγ-targeted regulatory function. Cotransfection of HEK293T cells with RORγ and a reporter construct containing either the G6Pase or FGF21 promoter upstream of a luciferase reporter gene resulted in transcriptional activation upon treatment with 5 μM SR1078 or Gala-SR (* represents the comparison with DMSO, *P<0.05, **P<0.01, n=4).

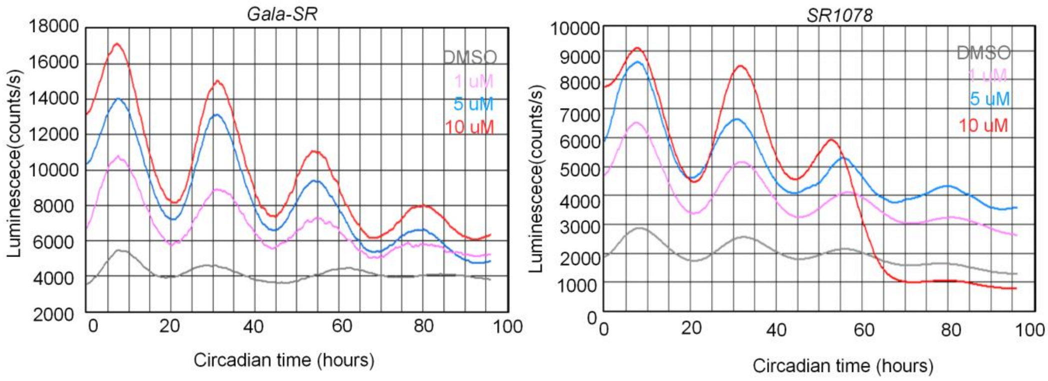

Figure S30. Gala-SR enhances circadian rhythm *in vitro*. Bioluminescence recordings were performed in *Bmal1-dLuc* U2OS cells treated with either Gala-SR (left) or SR1078 (right). (at concentrations of 1～10 μM), or DMSO treatment used as a control.

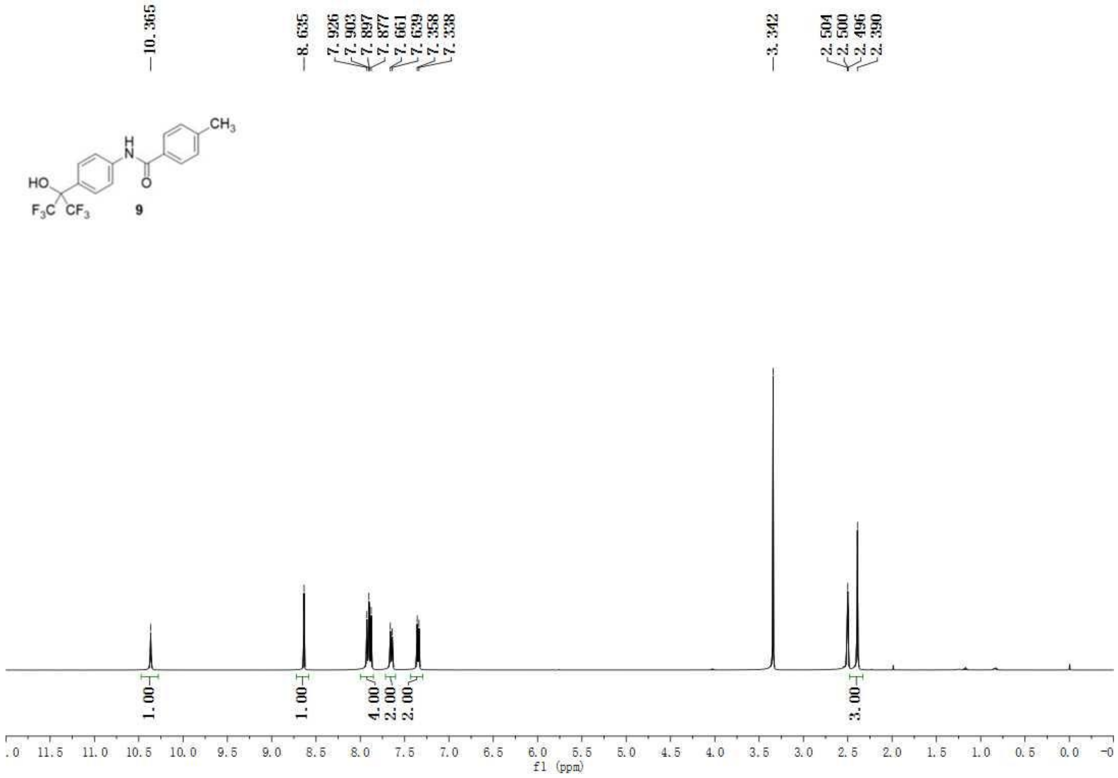

Figure S31. ^{1}H NMR (400 MHz, DMSO-*d6*) spectrum of **9**

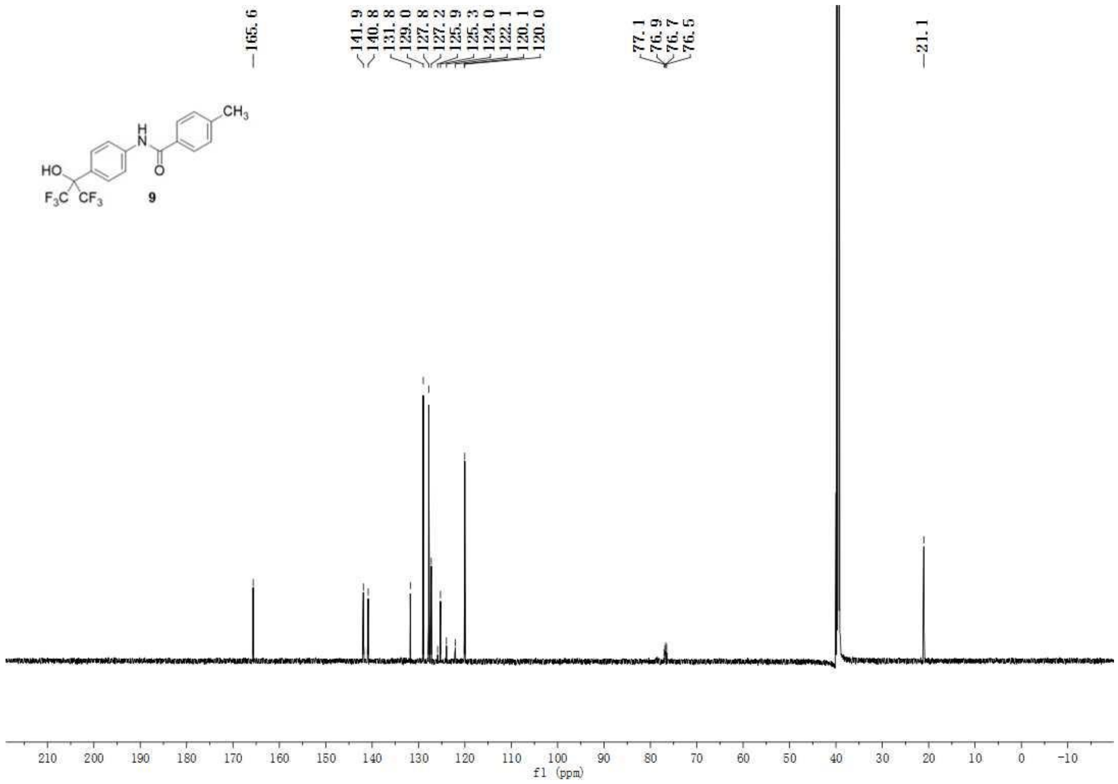

Figure S32. ^{13}C NMR (150 MHz, DMSO-*d6*) spectrum of **9**

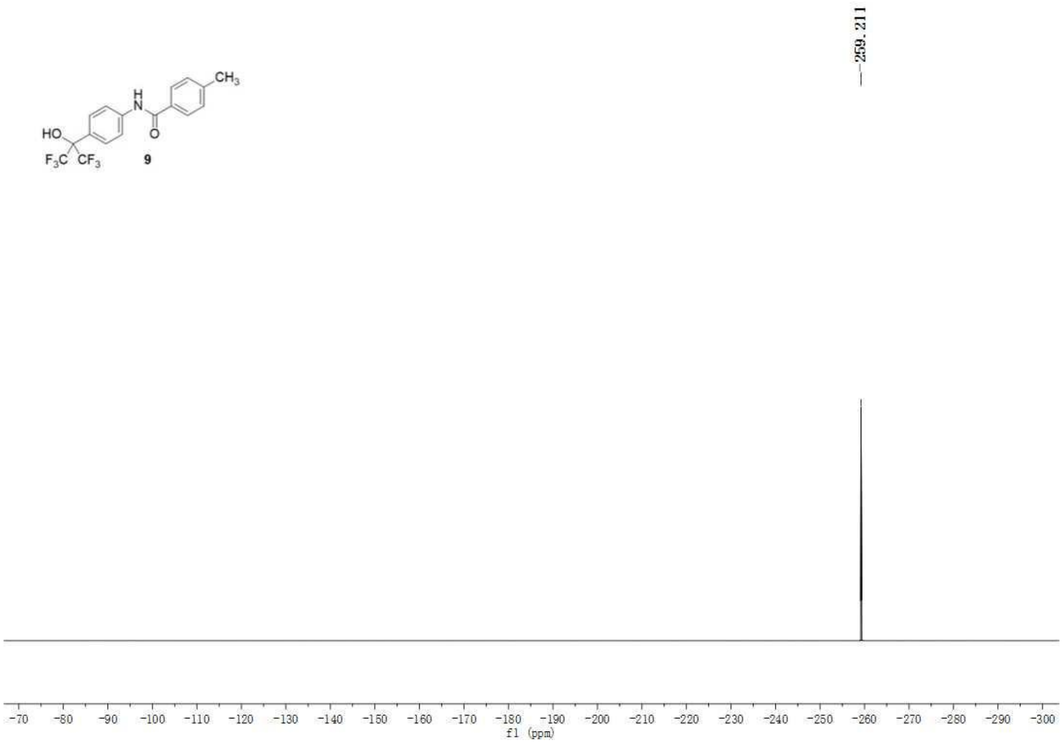

Figure S33. ^{19}F NMR (565 MHz, DMSO-*d*6) spectrum of **9**

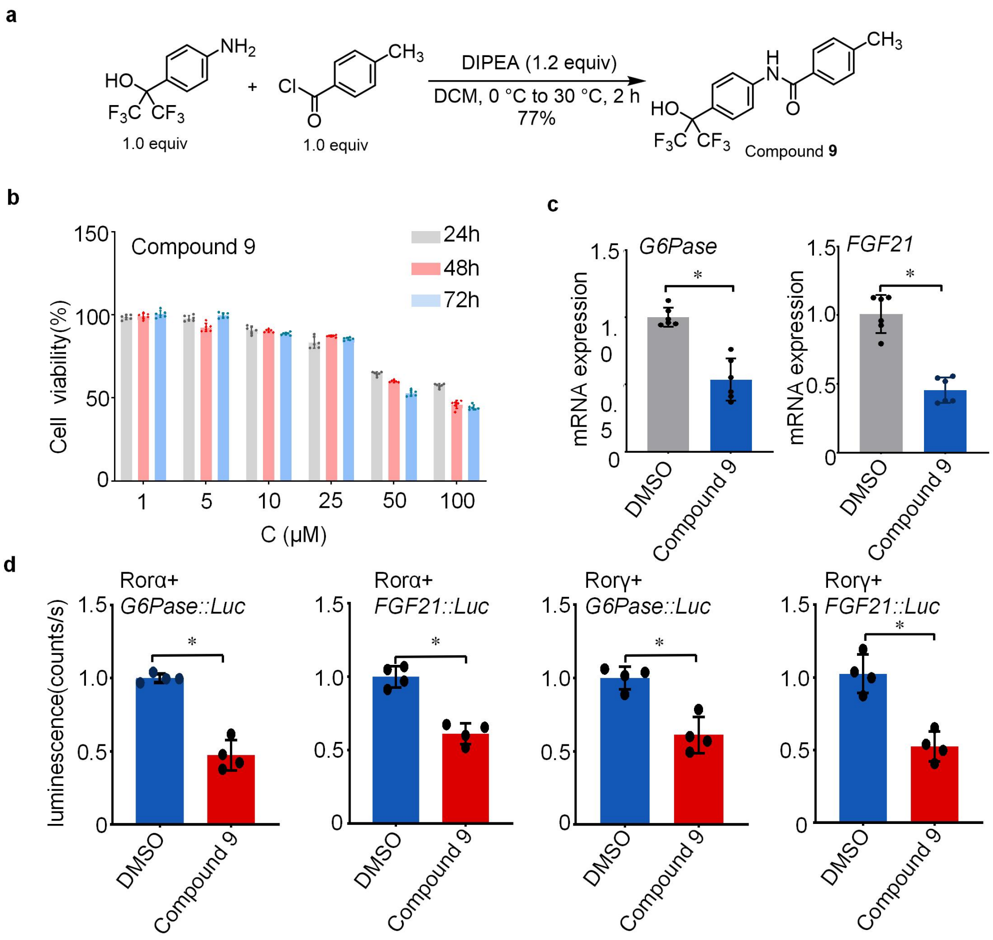

Figure S34. Exploration of the inhibitory effect of compound 9 on the targeting function of RORs. **a.** Synthetic route of compound 9. **b.** Cytotoxicity assessment of compound 9 by CCK-8 assay. **c.** qPCR analysis of downstream target genes (*FGF21* and *G6Pase*) in compound 9-treated HepG2 cells(n=6). **d.** Cotransfection of HEK293T cells with RORα or RORγ and reporters containing the *FGF2*1 or *G6Pase* promoter upstream of luciferase resulted in transcriptional stimulation after treatment with 5μM each of Gala-SR and SR1078. (* represents the comparison with DMSO*P<0.05, n=4).

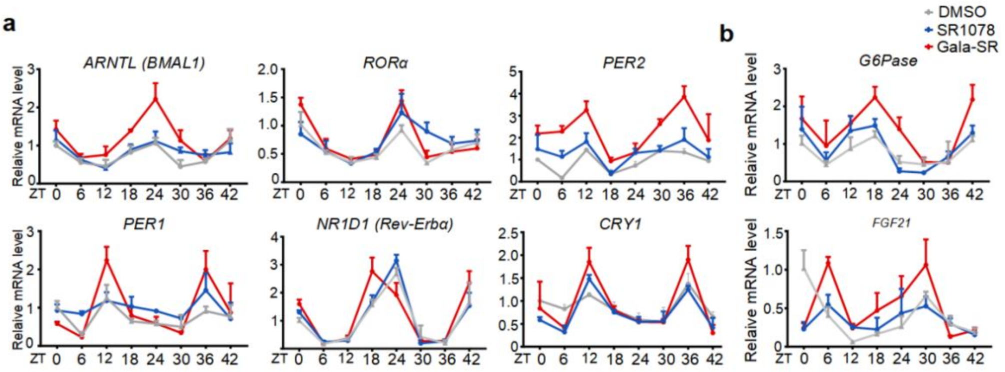

Figure S35. Impact of Gala-SR on circadian clock gene expression in periodontal ligament stem cells. qPCR analysis of core clock genes at multiple time points in synchronized hPDLSCs treated with 5 μM SR1078 or Gala-SR.

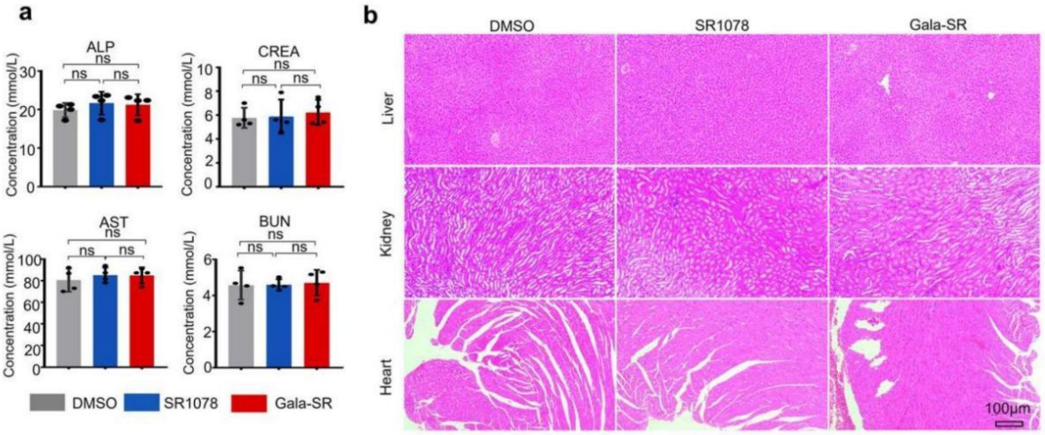

Figure S36.Toxicity and safety evaluation *in vivo*.**a.** After intraperitoneal injection of Gala-SR and SR1078 for 2 weeks, mouse serum was collected to measure liver function markers (ALT, AST, CREA, and BUN. ns, not significant) to assess the drugs' impact on liver function. **b**. HE staining was performed on liver, kidney, and heart tissues from mice after 2 weeks of intraperitoneal injection with Gala-SR and SR1078.

Reference

[1] Wang, Y.; Kumar, N.; Nuhant, P.; Cameron, M. D.; Istrate, M. A.; Roush, W. R.; Griffin, P. R.; Burris, T. P. *ACS Chem. Biol*. 2010, *5*, 1029.

[2] Elferink, H.; Titulaer, W. H. C.; Derks, M. G. N.; Veeneman, G. H.; Rutjes, F. P. J. T.; Boltje; T. J. 10.1002/chem.202103910.

[3] Deobald, A. M.; Camargo, L. R. S.; Diego Alves, Zukerman-Schpector, J.; Corrêa, A. G.; Paixão, M. W. *Synthesis*, 2011, 24, 4003.

[11] A. Herxheimer, K. J. Petrie, Cochrane Database Syst Rev 2002, (2), CD001520, https://doi.org/10.1002/14651858.CD001520.

[12] D. M. Minich, M. Henning, C. Darley, M. Fahoum, C. B. Schuler, J. Frame, Nutrients 2022, 14 (19), https://doi.org/10.3390/nu14193934.

[13] Z. Menczel Schrire, C. L. Phillips, J. L. Chapman, S. L. Duffy, G. Wong, A. L. D'Rozario, M. Comas, I. Raisin, B. Saini, C. J. Gordon, A. C. McKinnon, S. L. Naismith, N. S. Marshall, R. R. Grunstein, C. M. Hoyos, J Pineal Res 2022, 72 (2), e12782, https://doi.org/10.1111/jpi.12782.

[14] Z. Chen, S. H. Yoo, J. S. Takahashi, Cell Mol Life Sci 2013, 70 (16), 2985, https://doi.org/10.1007/s00018-012-1207-y.
