# Supplementary material for: Galactose-modified small molecule modulator targets RORα to enhance circadian rhythm and alleviate periodontitis-associated alveolar bone loss
Source: Bone Res. 2025 Oct 30;13:91. doi: 10.1038/s41413-025-00445-w (PMC12575728; doi:10.1038/s41413-025-00445-w)
Supplement: Supplementary file 2 — Supporting material-BONERES-04018R [file 41413_2025_445_MOESM2_ESM.docx]

**Table S1**. **Pharmacokinetic parameters of SR1078 and SR1078 following intraperitoneal injection and intravenous injection of the compounds at a dose of 10 mg/kg in mice.**

| PK parameters | Gala-SR-IP | SR1078-IP | Gala-SR-IV | SR1078-IV |
| --- | --- | --- | --- | --- |
| t1/2(hr) | 3.43 | 0.97 | 2.49 | 0.98 |
| T_max_(hr) | 0.5 | 0.5 | 0.5 | 0.5 |
| C_max_(ng/mL) | 1313.1 | 1811.68 | 4992.46 | 7127.36 |
| AUC(h*ng/mL) | 6077.31 | 1909.62 | 8176.18 | 2542.41 |
| MRT_Inf__obs(hr) | 4.50 | 1.194 | 2.97 | 0.487 |
| AUC/D(h*kg*ng/mL/mg) | 607.73 | 190.962 | 817.62 | 254.24 |
